# Arbuscular Mycorrhizal Symbiosis Leads to Differential Regulation of Drought-Responsive Genes in Tissue-Specific Root Cells of Common Bean

**DOI:** 10.3389/fmicb.2018.01339

**Published:** 2018-06-21

**Authors:** Gustavo H. Recchia, Enéas R. Konzen, Fernanda Cassieri, Danielle G. G. Caldas, Siu M. Tsai

**Affiliations:** Laboratory of Molecular and Cellular Biology, Center of Nuclear Energy in Agriculture, University of São Paulo, Piracicaba, Brazil

**Keywords:** arbuscular mycorrhizal fungi, *Phaseolus vulgaris*, water deficit event, root, RNA-Seq, laser-capture microdissection, aquaporins

## Abstract

Arbuscular mycorrhizal fungi (AMF) colonization in plants promotes both local and systemic changes in the gene expression profiles of the host that might be relevant for drought-stress perception and response. Drought-tolerant common bean plants (cv. BAT 477), colonized by a mixture of AMF (*Glomus clarum*, *Acaulospora scrobiculata*, and *Gigaspora rosea*), were exposed to a water deprivation regime of 96 h during pre-flowering. Root transcriptomes were accessed through RNA-Seq revealing a set of 9,965 transcripts with significant differential regulation in inoculated plants during a water deficit event, and 10,569 in non-inoculated. These data include 1,589 transcripts that are exclusively regulated by AMF-inoculation, and 2,313 under non-inoculation conditions. Relative gene expression analyses of nine aquaporin-related transcripts were performed in roots and leaves of plants harvested at initial stages of treatment. Significant shifts in gene expression were detected in AM water deficit-treated roots, in relation to non-inoculated, between 48 and 72 h. Leaves also showed significant mycorrhizal influence in gene expression, especially after 96 h. Root cortical cells, harboring or not arbuscules, were collected from both inoculation treatments through a laser microdissection-based technique. This allowed the identification of transcripts, such as the aquaporin *PvPIP2;3* and *Glucan 1,3 β-Glucosidase*, that are unique to arbuscule-containing cells. During the water deficit treatment, AMF colonization exerted a fine-tune regulation in the expression of genes in the host. That seemed to initiate in arbuscule-containing cells and, as the stressful condition persisted, propagated to the whole-plant through secondary signaling events. Collectively, these results demonstrate that arbuscular mycorrhization leads to shifts in common bean’s transcriptome that could potentially impact its adaptation capacity during water deficit events.

## Introduction

The great majority of plants are capable of establishing symbiotic relationships with arbuscular mycorrhizal fungi (AMF) that colonize root cortical cells forming intracellular structures named arbuscules (Parniske, 2008). AMF form an extensive hyphal network in the soil that heads through the roots depletion zone, therefore becoming able to acquire nutrients (mainly phosphates) and water in a more efficient way than the root system alone (Smith and Smith, 1990; Rapparini and Peñuelas, 2014). In return, AMF take up photosynthetically fixed carbon from plants and translocate it to their external mycelium mainly through synthesized lipid and glycogen (Bago et al., 2003). Besides, the ancient 400 million years’ history of co-evolution (Redecker et al., 2000), combined with the ability of around 80% of angiosperms to sustain this mutualistic relationship, suggests that plants might be able to sustain a wide system of genetic reprogramming related to the endosymbiotic process (Balestrini et al., 2007; Fiorilli et al., 2009; Gomez et al., 2009; Guether et al., 2009; Hogekamp et al., 2011; Gaude et al., 2012; Oldroyd et al., 2014).

Long-term climate changes have led to a global scale increase in the number of drought stress events in plants, and together with the constant population growth and consequent expansion of agricultural practices into marginal areas, have been generating more unstable production, therefore, severely diminishing food stocks all over the world (Parry et al., 2007). Water deficit is one of the most common abiotic stress factors that affect plants, directly interfering on its normal development and growth and having a major adverse effect on productivity (Colmenero-Flores et al., 1997). The development of new technologies to engineer drought tolerance in plants has huge economic importance (Umezawa et al., 2006) and the use of AMF inoculation on annual cultures with the objective of improving plants properties has emerged in the last few years as a more sustainable practice (Azcón-Aguilar and Barea, 1997; Barea et al., 2005; Cozzolino et al., 2013).

The possibility of increasing drought tolerance in plants through AMF inoculation has been extensively investigated (Augé, 2001; Bárzana et al., 2014, 2015; Rapparini and Peñuelas, 2014; Saia et al., 2014). The symbiosis protects plants against the adverse impacts of water deficit mainly through the maintenance of leaf water potential, relative water content (RWC), stomatal conductance, CO_2_ assimilation, photosystem II efficiency, and transpiration (Augé, 2001; Ruiz-Lozano, 2003; Sánchez-Blanco et al., 2004; Bárzana et al., 2012; Lee et al., 2012). This is achieved through a better nutritional status, that secures a more sustainable physiology throughout plant development, and the induction of drought avoidance mechanisms consisting on the preservation of an adequate plant hydration status that helps keeping constant cellular turgor pressure on leaves during low water potential (Augé, 2001; Augé et al., 2004; Rapparini and Peñuelas, 2014). Additionally, the hyphal natural characteristics, such as a diameter twice smaller the average root size (2–5 μm), allows the fungus to extend its network throughout the water depletion zones and inside thinner pores that might retain water through the dryness process (Ruiz-Lozano, 2003; Miransari et al., 2007; Rapparini and Peñuelas, 2014).

The interaction with AMF may result in both local and global changes in the expression profiles of genes that are vital for both biotic and abiotic resistance responses in plants (Porcel et al., 2012). Molecular techniques have been applied, and a significant number of transcripts have been described as differentially regulated in AM plants during water deficit. Two Δ-pyrroline-5-carboxylate synthetase genes, *gmp5cs* from *Glycine max* e *lsp5cs* from *Lactuca sativa*, and three genes encoding for dehydrins, *gmlea8* and *gmlea10* (*G. max*) and *lslea 1* (*L. sativa*), were down-regulated in the roots of the respective plants under AM inoculation (Porcel and Ruiz-Lozano, 2004; Porcel et al., 2005). In *Phaseolus vulgaris* roots colonized by *Glomus intraradices*, drought treatment increased the expression of the aquaporin *PvPIP1;1* gene, drastically diminished the expression of the *PvPIP1;2* and *PvPIP1;3* genes, and did not change the expression of the *PvPIP2;1* gene. Non-AM control plants revealed higher gene expression of the analyzed transcripts during drought, except for *PvPIP1;2* (Aroca et al., 2006).

Despite common bean has great importance in human populations diet of developing regions such as Latin America and Africa, it is still a low scale production culture, mostly performed in small-farming systems, with minimum technological input, being 60% of it relegated to regions that suffer with continuous or intermittent drought events during the year (CIAT, 1989; Thung and Rao, 1999). The highly diverse conditions under which common beans are cultivated, combined with local preferences for specific seed traits, have led to difficulties in establishing efficient breeding programs that are able to combine both improved performances under drought stress with enhanced growth and production (Graham and Ranalli, 1997; Beebe et al., 2009).

BAT 477, a type III-growth habit drought-resistant line, has deep rooting ability and high water absorption efficiency (Sponchiado et al., 1989), serving well as a biological model for drought tolerance in common bean studies. Terminal drought simulation studies in soil tubes indicated that BAT 477 has the ability to grow tap roots under drought conditions and inhibit lateral root growth (Rao et al., 2006). Although great advance has been made in root development and architecture, soil fertility in tropical soils is poor, mainly regarding phosphorous availability, and this factor can limit vegetative development and root growth (Thung and Rao, 1999). Therefore, since a shallow and abundant root system is more effective for nutrient acquisition, and a deep root system is more efficient for water absorption, the combination of tolerance with these two traits can be a great challenge (Beebe et al., 2009). Thus, the more efficient establishment of symbiotic relationships with soil microbiome in the roots, such as AMF, may provide an interesting strategy for overcoming this issue.

Since common beans have a simpler genomic structure (2*n* = 22), the culture fits well as a model for other more complex tropical legume crops, like soybean (*G. max*) (Galeano et al., 2008; McConnell et al., 2010). In this study, the symbiotic interaction established between common bean plants (BAT 477, drought-tolerant) and a mixture of three AMF strains (*G. clarum*, *A. scrobiculata*, and *G. rosea*) was explored to further evaluate the influence exerted by the colonization process over plants transcription profiles of important genes related to drought-stress response and adaptation. A whole-transcriptome sequencing (RNA-Seq) approach was adopted, and after careful statistical comparisons, provided an interesting set of candidate genes for a more detailed characterization of our model of study. A time-course experiment was conducted, enabling the detection of shifts in the relative expression of nine aquaporins, as evaluated through RT-qPCR considering whole-roots and -leaves harvested at 24, 48, 72, and 96 h of both water deficit and inoculation treatments. This step allowed the identification of a mycorrhizal influence over common bean transcriptome that varies both at a time and spatial configuration. Moreover, root cortical cells (harboring or not arbuscules) were obtained from water deficit treated plants through laser-capture microdissection microscopy (LCM), and the relative expression of 21 transcripts was compared between the different cell populations. This provided additional evidences for the occurrence of a site-specific modulation mechanism of the host transcriptome during AMF colonization.

## Materials and Methods

### Biological Material and Water Deficit Assay

BAT 477 seeds were superficially sterilized by rinsing them for 1 min in sodium hypochlorite 10% and washing three times with distilled water. Open-pot cultures were obtained by growing *Brachiaria ruziziensis* plants in a sand:vermiculite (2:1) substrate inoculated with the AMF mixture. The mycorrhizal inoculum for each endophyte (*G. mossae*, *A. scrobiculata*, and *G. rosea*) consisted of 50 ml soil containing spores, mycelium, and 75% infected root fragments obtained from open-pot cultures of *Brachiaria decubens*.

BAT 477 seeds were placed in 8 liter pots containing a mixture of sand:vermiculite (2:1), both pre-washed and sterilized at 120°C and 1 atm for 30 min. AMF inoculation was performed with 50 ml of a mixture containing an average 30 spores per gram, mycelia and hyphal structures of the AMF mixture obtained from the open-pot culture; non-inoculated treatments received 50 ml of a pre-sterilized mixture.

Experiments were conducted at greenhouse conditions (24–27°C). Four treatments with 18 replicates were applied: (i) AMF-inoculated plants under water deficit (Myc+/Strss); (ii) AMF-inoculated plants with regular irrigation (Myc+/Ctrl); (iii) non-inoculated plants under water deficit (Myc-/Strss); and (iv) non-inoculated plants with regular irrigation (Myc-/Ctrl). Plants were kept under a regular irrigation regime of approximately 200 ml distilled deionized water every 2 days. Irrigation was performed in order to keep plants at 80% of field capacity, which corresponds to 21–24% of soil moisture content that was monitored using a digital thermo-hygrometer equipped with a sensing probe.

Irrigation was kept constant until plants reached the pre-flowering developmental stage (R5), when plants dependence under stored soil–water intensifies and effects of terminal drought on subsequent flowering and pod-filling becomes more limiting (Rosales-Serna et al., 2004). Water deficit treated plants had water supply fully suspended during 96 h, thus simulating a severe water deficit event. Soil water moisture content (%) was monitored during the whole period. Three applications of nutrient solution (200 ml) were performed, after 10, 17, and 21 days, respectively, following Hoagland’s (Hoagland and Arnon, 1950) formulation. Inoculated plants received a modified formulation (0.25 mmol/l of KH_2_PO_4_ solution, added 1 mmol/l KNO_3_) in order to prevent an impairment in root colonization due to a high phosphate concentration (Balzergue et al., 2013). Plants harvestings, consisting of three randomly chosen biological replicates from each treatment, started after 24 h of water deficit treatment, being repeated after 48, 72, and 96 h.

After 96 h, from the nine biological replicates left in each treatment, three were randomly selected for water deficit assay validation, three were stored at -80°C for total RNA extraction and three applied for microscopy. Following, gas-exchange measurements were conducted in fully expanded intermediate leaves of three plants of each treatment (between 11:00 a.m. and 01:30 p.m.) with a gas-exchange system (IRGA LI-6400XT; Li-Cor). Leaves were first equilibrated at a photon density flux of 1000 mmol m^-2^ s^-1^ for at least 20 min. After this, photosynthesis was induced with 1000 mmol photons m^-2^ s^-1^ and ∼386 mmol mol^-1^ CO_2_ surrounding the leaf. Leaf temperature was maintained at 28°C, and the leaf-to-air vapor pressure deficit was kept between 1 and 1.3 kPa. These conditions were kept constant for the determination of net photosynthetic rate (*A*_N_), transpiration rate (*E*), stomatal conductance (*g*_s_), and water-use efficiency (WUE). WUE was calculated as the ratio between photosynthesis rate and stomatal conductance (*A*_N_/*g*_s_) (Dewar, 1997).

Moreover, three completely and recently expanded trifoliates from each plant were harvested and weighed for RWC determination according to Barrs and Weatherley’s (1962) instructions and following the equation: RWC (%) = [(Fw-Dw)]/[(Tw-Dw)] ^∗^ 100.

The root length density (RLD), the length of roots per unit volume of substrate, was measured by placing the whole root organs (three biological replicates for each treatment) on a board covered by a squared paper (1 × 1 cm). Boards were scanned, and length measurements were taken using ImageJ 1.46r software^1^. RLD was obtained by the average length of root segments (cm) proportional to the volume occupied by the substrate in the respective pot (cmł) – given by the calculation of the volume of a circular truncated cone. Total leaf and root dry mass (LDM and RDM) were determined by drying samples in a ventilated oven at 60°C (∼48 h) until reaching constant weight.

Mycorrhizal colonization rates MCR(%) were estimated from 1 g of roots obtained from each triplicate and treatment and estimated following previous protocol (Brundrett et al., 1996), using a stereo microscope system (Leica EZ4 HD – Leica Microsystems).

The JMP – Statistical Discovery^TM^ from SAS software was used for an analysis of variance (ANOVA) to discriminate the effects of the water deficit event and AMF (+ or -) treatments. To perform the ANOVA, we also acknowledged prior steps toward the verification of normality and variance homogeneities of the data. Significant differences encountered through the ANOVA (*p* < 0.05) were further accessed for mean comparisons among the treatment combinations, using Tukey’s test with Laercio package from R (v.i.3.2.2)^2^.

### Sample Collection and RNA Preparation for RNA-Seq Analysis

Whole root tissues were retrieved from the last three biological replicates in each treatment, ground in liquid nitrogen, and stored at -80°C prior to RNA extraction. Total RNA was isolated with Trizol (Trizol Reagent^®^ LS, Invitrogen) following manufacturer’s recommendations. RNA samples were quantified and assessed for quality and molecular integrity using the RNA 6000 Nano LabChip^®^ Kit (Agilent Technologies) through the Agilent 2100 Bioanalyzer (Agilent Technologies) system following manufacturer’s instructions. RNA samples were prepared for sequencing using the TruSeq^TM^ RNA Sample Preparation Kit v2 (Illumina^®^ Sequencing). The library clusterization was conducted using TruSeq^®^ PE Cluster Kit v3 (para cBot – HiSeq/HiScan SQ) and sequencing was performed with TruSeq^TM^ SBS kit v3 kit (for HiSeq/HiScan SQ) following manufacturer’s instructions in a HiScan^TM^ Sq System (Illumina^®^).

### Sequences Assembly and Data Analysis

Paired-end sequencing raw data were obtained through the CASAVA 1.8.2 (Illumina^®^) software. Reads were trimmed for adaptors and vectors sequences and purified from low-quality sequences using the SeqyClean v.1.9.7 software^3^ with cutoff of 24QScore and Univec database for contaminants^4^. Transcriptome mapping was conducted using the common bean reference genome data publicly available on Phytozome database v.11.1^5^ (Schmutz et al., 2014). Paired-end filtered reads are available at NCBI’s Sequence Read Archive Public Database^6^, individual temporary submission IDs are available (**Supplementary Table S1**).

Reference genomes were indexed using Bowtie 2 v.2.1.0 software tool (Langmead and Salzberg, 2012). Individual mappings of the samples were achieved following Bowtie2 v2.1.0 software package taking indexed genomes and individual annotation files (public available by the Joint Genome Institute^7^) as references. The transcript assembly was achieved following the Cufflinks v2.1.1 package tools pipeline using the RABT option (Trapnell et al., 2010). The individual assemblies were merged using the function cuffmerge from Cufflinks. Raw reads abundance estimation counts were obtained through HTSeq-count v.05.4.p2 script^8^ through the annotation file obtained from Cufflinks assembly.

Differentially expressed genes (DEGs) between the optimal irrigation and water deficit treated samples, as well as inoculated and non-inoculated plants both under water deficit, were identified using the DESeq2 package from R/Bioconductor (Love et al., 2014). The -in function “estimate Size Factors” was used to obtain the normalized counts, i.e., baseMean values, which are the number of reads divided by the size factor or normalization constant. Transcripts with baseMean less than five considering all samples were removed to avoid artifacts due to low coverage. The method used to test for differential expression was the negative binomial distribution, followed by the FDR correction (<0.05) (Benjamini and Hochberg, 1995).

Putative DEGs transcripts annotation were obtained through *blastx* function with cutoff of 1e-10 using the blast+ package^9^. The green plant public database of NCBI^10^ was used as reference. Functional classification inside Gene Ontology terms^11^ and Pfam was obtained through Blast2GO software (Conesa et al., 2005). Hit files obtained through blastx were annotated by the GOSlim function followed by the enrichment of genes inside the most representative ontology terms in each of the four treatments using a *Fisher-2 tailed test* with FDR (<0.01) through Blast2GO software. Briefly, the four sets of DEGs retrieved from each differential expression statistical analysis were compared against the complete set of annotated transcripts of common bean. For those ontology terms where the number of represented DEGs were superior to that of the reference set, the term was considered *over-represented* for that particular treatment; and for those terms where the number of represented DEGs were inferior, it was considered *under-represented*.

Heat maps were constructed based on the means of normalized read counts assigned to individual transcripts in each sample after differential gene expression analysis. The Heatmap.2 function from gplots (v 2.12.1) R package (R i386 3.2.0) was used for designing the heat maps.

### Temporal Analysis of Aquaporin-Genes Expression: RNA Extraction, RT-qPCR Assay, and Data Analysis

Total RNA was extracted from leaves and roots harvested in all time-periods of water deficit and control treatments described before. RNA extraction was performed by following the TRIzol^®^ Reagent kit (Invitrogen) manufacturer’s instructions from 50 mg of grinded frozen tissues as described previously. Total RNA extract was diluted in 20 μl 0.1% DEPC-water. The RNA samples were quantified by spectrophotometry at 260 and 280 nm wavelengths using NanoDropTM 2000c, and RNA quality was verified by electrophoresis on a 1.6% agarose gel in TAE buffer. Purified total RNA was guaranteed by carrying out DNAse treatment using a DNAse1 RNAse-free kit (Fermentas), following the manufacturer’s instructions. For RT-qPCR assay, 100 ng of DNAse-treated total RNA was used for cDNA synthesis using the Maxima^TM^ First Strand cDNA Synthesis Kit for two step RT-qPCR kit (Fermentas^TM^) following the manufacturer’s guidance.

Reactions were carried out on a StepOnePlus^TM^ Real Time PCR System (Applied Biosystems) using the Maxima^®^ SYBR Green/ROX qPCR Master Mix kit (Fementas^TM^), following manufacturer’s instructions. Each reaction was prepared to a final volume of 10 μl: 0.25 μM of each primer, 100 ng of cDNA, 2× of SYBR Green Mix. R reactions conditions: 10 min at 42°C; 10 min at 95°C; 40 cycles of cDNA amplification for 15 s at 95°C, 20 s at 59°C, and 30 s at 72°C with fluorescent signal recording. At the end, a final step of 15 s at 95°C, 1 min at 60°C, and fluorescence measured at each 0.7°C variation (from 60 to 95°C). Three reference genes were validated for our conditions and set as internal normalizers for relative expression assays, *skip16* and *IDE* for roots, and *Ubq* and *IDE* for leaves (Borges et al., 2012). No-template control reactions were performed for each pair of primers by omitting cDNA template and completing the final volume of the reaction with water. Experimental design consisted of three technical replicates for each biological triplicate (3 × 3) per cell-type analyzed and amplified transcript.

Raw data (not baseline corrected) of fluorescence levels were submitted to LinRegPCR software (Ruijter et al., 2009), for baseline correction and linear regression analysis on each amplification curve. The optimal set of data points (window-of-linearity) was defined to allow the calculation of the threshold and quantification cycle (Cq) values. Furthermore, the efficiency was calculated based on slope of the line (*E* = 10^slope^), considering an ideal value range (1.8 ≤*E* ≤ 2) and correlation (*R* ≥ 0.995).

Relative expression data were obtained by REST software (Pfaffl, 2001) using average values of efficiency and Cq of target and reference genes. This software compares control and treatment Cq values to obtain the concentration of expression (*C*), where *C = E*^meanCq(Control)^ ^-meanCq(treatment)^; then, it calculates the relative expression (RE) ratio where RE = *C*_target gene_/geometric average *C*_reference gene_; and performs a pairwise fixed reallocation randomization test (bootstrap = 2,000 permutation) to obtain *p-*values.

### Sample Preparation for Light-Microscopy Visualization

Root segments (1 cm) were harvested from 96 h AMF-inoculated water deficit event treated plants (Myc+/Strss) and fixed in a Karnovsky modified solution (2% glutaraldehyde, 2% paraformaldehyde, 1 mM CaCl_2_, 50 mM sodium cacodylate pH 7.2) at 4°C for 48 h. After, samples were dehydrated in ascending ethanol series (35–100%) and infiltration was performed slowly in ethanol. Samples were embedded in Historesin (*HistoResin Mounting Media kit – Leica Helderberg*) following manufacturer’s instructions. Tissue section (5 μm thick) were obtained in a RM2155 microtome (Leica^®^ Germany) and mounted in glass blades. Staining was conducted as follows: 2 min in pre-heated (40°C) 1% acid-Fuchsin, washed in tap water, 5 min in 0.05% Toluidine-blue, and washed in tap-water. Tissue sections were observed in a Leica LMD 7000 (Leica Microsystems^®^) microscope and images were processed using LAS.v.3.8 software.

### Microscope-Based Laser-Capture Microdissection and Total RNA Extraction

Root fragments (1 cm) were collected from the three last biological replicates of both 96 h water deficit treatments (Myc+/Strss and Myc-/Strss) and fixed in 10% paraformaldehyde solution (diluted in 0.1% DEPC-treated distilled water) at 4°C for 24 h. Root fragments were retrieved from the same three biological replicates selected for the MCR(%) analysis. Transversal root sections were paraffin embedded following (Ludwig and Hochholdinger, 2014) protocol. Sections were dehydrated in an ethanol graded series (70, 80, 90, and 100%), followed by an ethanol and xylol series (3:1; 1:1; 1:3; and 100% ethanol) for 4 h at room temperature; samples were embedded through a gradient of dehydration solution and paraffin (3:1; 1:1; 100% paraffin) at 58°C by 4 h each. Transversal histological sections were 10 μm thick and mounted into PEN slides (Leica Microsystems^®^) and stored at -80°C. A minimum set of 24 blades were mounted for each biological triplicate consisting of root segments harvest from three different plants and relative to each treatment, Myc+/Strss and Myc-/Strss (24 × 3 × 2).

Prior to laser microdissection, slides were gradually thawed (20 min at -20°C and 15 min at 4°C), followed by sample paraffin removal through dehydration at room temperature consisting of total immersion on 100% xylol for 10 min, 1 min in 100% ethanol, additional ethanol graded series (95% and 70%) for 30 s each and 1 min in 0.01% DEPC-treated distilled water. Staining followed a modified version of Feder and O’Brien (1968) protocol by immersion on 1% acid-Fuchsin solution pre-heated at 40°C for 2 min, washed in water, dropped in 0.05% Toluidine blue solution for 5 min, and again washed in water. A second dehydration step was performed by dropping samples into 95% ethanol for 90 s, 100% ethanol for 1 min, and 100% xylol for 10 min.

**FIGURE 1:**
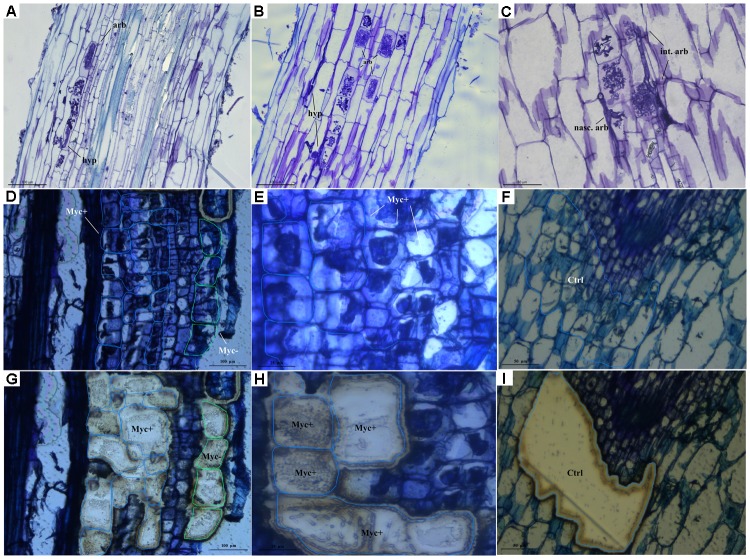
Histological section analysis. **(A–C)** Light microscopy of Historesin-embedded sections of water deficit treated AMF-inoculated roots. **(D–I)** Microscope-based laser-capture microdissection. **(D)** Arbusculated root-cortical cells (blue line) and neighboring non-arbusculated cortical cells (green line). **(E)** Arbusculated root cortical cells. **(F)** Root cortical cells from non-inoculated plants. **(G, H)** Tissue sections from AMF-inoculated water deficit treated roots after dissection. **(I)** Tissue sections from non-inoculated water deficit treated roots after dissection. arb, arbuscules; hyp, AMF hyphal structures; int. arb, interconnected arbuscules; nasc. arb, nascent arbuscules; Myc+, arbusculated root cortical cells; Myc–, non-arbusculated neighboring cortical cells; ctrl, root cortical cells from non-inoculated roots.

Laser microdissection was performed using Leica LMD 7000 (*Leica Microsystems*^®^). Three cell populations were assessed for each biological triplicate: ctx+ – root cortical cells containing arbuscules and/or hyphal structures derived from Myc+/Strss treatments, ctx- – root neighboring cortical cells not containing arbuscules derived from Myc+/Strss treatments; and ctrl – root cortical cells derived from Myc-/Strss treatments (**Figure 1**). Each laser microdissection session consisted of around 1 million μm^2^ sections for each population type.

Total RNA extraction was performed using the *Arcturus*^®^ *PicoPure*^®^ *RNA Isolation kit* (Applied Biosystems^TM^) following manufacturer’s instructions. For each cell type, the average amount (ng/μl) and quality (λ) of total RNA extracted was: ctx+ – 15.14 ng/μl and 1.85 λ; ctx- – 12.96 ng/μl and 1.79 λ; ctrl – 13.6 ng/μl and 1.83 λ (data not shown). Purified total RNA was guaranteed by carrying out DNAse treatment using a DNAse1 RNAse-free kit (Fermentas), following the manufacturer’s instructions. For RT-qPCR assay, 100 ng of DNAse-treated total RNA was used for cDNA synthesis using the *Maxima^TM^ First Strand cDNA Synthesis Kit for two step RT-qPCR kit* (Fermentas^TM^) following the manufacturer’s guidance.

### RT-qPCR Primers Design

For temporal differential gene expression analysis, a total of nine aquaporin transcripts were selected: *PvTIP1;1*, *PvPIP1;1*, *PvPIP1;2*, *PvPIP1;3*, *PvPIP1;5*, *PvPIP2;1*, *PvPIP2;3*, *PvPIP2;5*, and *PvPIP2;6*. Primers for *PvPIP1;2*, *PvPIP1;1*, and *PvPIP2;5*, respectively, homologous to *PvPIP1;1*, *PvPIP1;3*, and *PvPIP2;1* were retrieved from Aroca et al. (2007). The six other transcripts were chosen based on sequence homology to previously described plant’s aquaporins responsive to AM-inoculation or to a water deficit event stress response (Uehlein et al., 2007; Alexandersson et al., 2010; Recchia et al., 2013).

For LCM/RT-qPCR analysis a set of 23 common bean transcripts were selected among the most up- and down-regulated DEGs contrasting Myc+/Strss and Myc-/Strss treatments. PRIMER 3 software (version 0.4.0^12^) was used for primers design considering the following parameters: product size range of 100–200 bp; primer size of 18–22 bp; primer Tm of 57–63°C. NetPrimer software was used for quality check^13^. The 23 primer pairs are described in **Supplementary Table S2**.

### Endogenous Control

The endogenous control was based on PCR amplification of AML1–AML2 primers described by Lee et al. (2008). This pair of primers targets a small subunit rRNA gene and was designed to amplify all subgroups of AMF (Glomeromycota), but excluding sequences from other organisms, therefore allowing rapid identification of potential AM genomic-DNA contamination in ctx- and ctrl cell samples. Total DNA was extracted from the three tissue samples using QIAamp DNA Micro Kit (QIAGEN^®^) following manufacturer’s instructions. AML1–AML2 PCR amplification reactions were conducted as follows: 10× of PCR buffer, 50 mM of MgCl_2_ (50 mM), 2.5 mM of dNTP’s mix, 2.5 mM of each primer, 20 ng/μl of cDNA; 0.2 μl of Taq Polymerase (50U/1 μl), in a 25 μl final volume; the amplification conditions were: 2 min at 94°C, 30 cycles of 30 s at 94°C, 40 s at 58°C, 55 min at 72°C; 5 min at 72°C. The products from amplification were visualized in a 1.2% agarose gel in 1× TSB buffer.

### RT-qPCR Assay and Data Analysis

Reactions were carried out on a StepOnePlus^TM^ Real Time PCR System (Applied Biosystems) using the *Maxima^®^ SYBR Green*/*ROX qPCR Master Mix* kit (Fementas^TM^), following manufacturer’s instructions. Reactions were prepared for a 10 μl final volume: 0.25 μM of each primer, 100 ng of cDNA, and 2× of SYBR Green Mix. Reactions conditions: 10 min at 42°C, 10 min at 95°C, 40 cycles of cDNA amplification for 15 s at 95°C, 20 s at 59°C, 30 s at 72°C with fluorescent signal recording. At the end, a final step of 15 s at 95°C, 1 min at 60°C, and fluorescence measured at each 0.7°C variation (from 60 to 95°C) was included to obtain the melting curve. Two reference genes were validated for our conditions and set as internal normalizers for relative expression assays, *skip16* and *IDE* (Borges et al., 2012). No-template control reactions were performed for each pair of primers by omitting cDNA template and completing the final volume of the reaction water. The experimental design consisted of three technical replicates for each biological triplicate (3 × 3) per cell-type analyzed and amplified transcript. Base line correction and linear regression of raw data, quantification cycle (Cq) values and efficiency calculations, and relative expression analysis were performed as previously described for time-shift aquaporin gene expression analysis.

## Results

### Plant Growth and Physiological Parameters

Soil moisture content (%) was monitored throughout the whole 96 h of water deprivation treatment (**Supplementary Figure S1a**). Pots from Myc+/Ctrl plants indicated a slightly superior soil moisture content (∼22.9%) than the Myc-/Ctrl (∼20.9%). For both Myc+/Strss and Myc-/Strss treatments, soil moisture content decreased during the whole period ranging from 21 to 6% for Myc+, and from 21.2 to 4.23% for Myc-. Roots mycorrhizal colonization rates (MCR%) (**Supplementary Figure S1b**) indicated a significant statistical difference (*p* < 0.05) between Myc+/Ctrl and Myc+/Strss, with 55.74% colonization for non-stressed control AM plants and 74.44% in water deficit treated AM plants.

**FIGURE 2:**
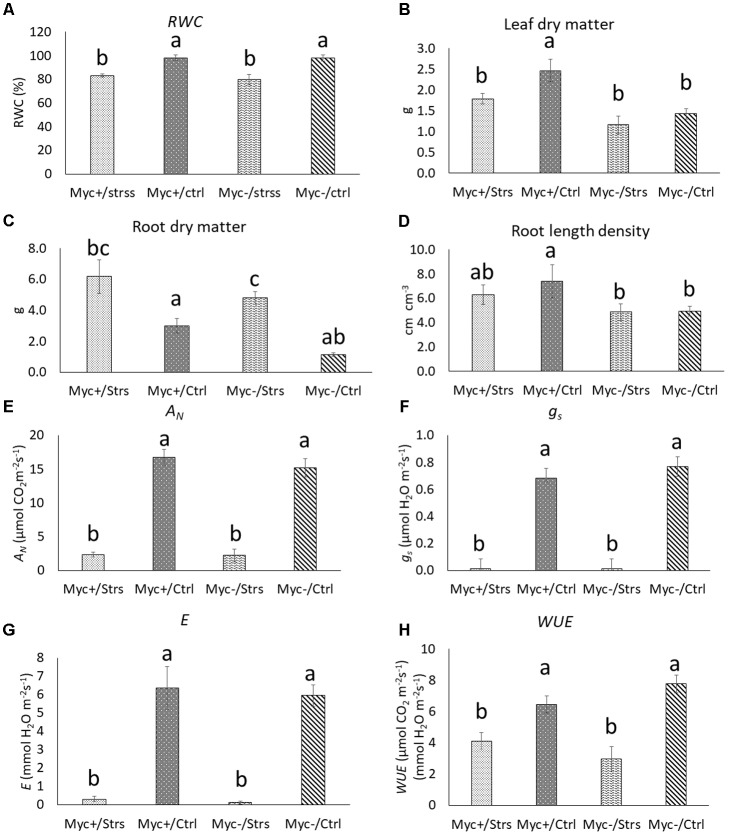
Physiological and growth parameters. **(A)** Relative water content (RWC) of leaf tissue; **(B)** leaf dry matter (LDM); **(C)** RLD; **(D)** root dry matter (RDM); **(E)** net photosynthetic rate (*A*_N_); **(F)** stomatal conductance (*g*_s_); **(G)** transpiration rate (*E*); **(H)** WUE. Myc+/Stress, AM plants under 96 h of water deficit; Myc+/Ctrl, AM plants under regular irrigation control; Myc–/Stress, no-AM plants under 96 h of water deficit; Myc–/Ctrl, no-AM plants under regular irrigation control. Bars represent standard deviation. Different letters indicate significant differences by Tukey’s test (*p* < 0.05).

The RWC quantification was performed to measure the plant water status of treated and control plants in terms of the physiological consequences of cellular water deficit. Statistical analysis of RWC data was able to distinguish the two groups, control and water deficit event treatments; however, no effect of AMF colonization was detected (**Figure 2A**). Additionally, plant growth parameters were accessed to further characterize the impact of AM-root colonization over common bean response and the adaptation to a water deficit event. The dry matter content of total leaves (LDM) and roots (RDM) was calculated. Regarding LDM, Myc+/Ctrl plants presented the highest dry matter accumulation (2.5 g), with no statistical differences detected among Myc+/Strss plants and both no-inoculated treated and control plants (**Figure 2B**). For RDM, no statistical significant differences were detected among the results (**Figure 2C**). RLD, the length of roots per unit volume of substrate, was measured with no statistical significant changes found between inoculated and non-inoculated plants, the only exception regarding a slightly increase in Myc+/Ctrl measurements (∼7.4 × 10^-4^ cm^-2^) (**Figure 2D**).

The physiological status of the plants from the experiment was also accessed with the measurement of photosynthetic rates. The net photosynthetic rate (*A*_N_), stomatal conductance (*g*_s_), transpiration rate (*E*), and WUE all differed (*p* < 0.05) among water deficit treated plants and the control (**Figures 2E–H**). No differences, however, were detected between inoculated and non-inoculated plants under water deficit, except for the WUE, which was significant, but comparatively, with only slight modification.

### Generation and Assembly of Transcript Reads

Twelve RNA-Seq libraries were constructed for paired-end sequencing consisting of biological triplicates from the four treatments: Myc+/Strss, Myc+/Ctrl, Myc-/Strss, and Myc-/Ctrl. The Illumina^®^ HiScan^TM^Sq sequencing of the 12 samples generated sequence reads of 101 bp long, with an average GC content of 45% and a phred value of 36. After sequences filtering, the average amount of reads for each dataset was: 21,611,562 for Myc+/Strss; 20,189,276 for Myc+/Ctrl; 22,664,734 for Myc-/Strss; and 20,247,646 for Myc-/Ctrl. The average amount of reads successfully mapped against *P. vulgaris* reference genome was: 68.32% for Myc+/Strss, 81.90% for Myc+/Ctrl, 85.50% for Myc-/Strss, and 86.25% for Myc-/Ctrl (**Supplementary Table S1**).

### Differential Gene Expression Analysis

Individual gene and transcript annotation “track” files, retrieved for each triplicate, were merged per corresponding treatment, and tested for differential expression due to the “treatment” factor. Comparisons were made between group pairs and four statistical results were retrieved: Myc+/Ctrl vs. Myc+/Strss; Myc-/Strss vs. Myc+/Strss; Myc-/Ctrl vs. Myc+/Ctrl; and Myc-/Ctrl vs. Myc-/Strss (**Supplementary Table S3**).

The comparison that returned the highest amount of statistically significant (*p*-value with FDR < 0.05) DEGs, including transcripts isoforms, was related to non-inoculated plants, well-watered against 96 h’ water deficit treated (Myc-/Ctrl vs. Myc-/Strss), with a total of 10,569 DEG – 5,044 of them up-regulated under water deficit exposure and 5,525 down-regulated by the water deficit event. Inoculation treatments, control against water deficit (Myc+/Ctrl vs. Myc+/Strss), also produced a substantial level of differential expression, with 9,965 DEG – 4,668 were up-regulated by the water deficit treatment and 5,297 down-regulated (**Supplementary Table S3**).

For the differential gene expression analyses comparing non-inoculated against AMF-inoculated treatments, control (Myc-/Ctrl vs. Myc+/Ctrl) and water deficit (Myc-/Strss vs. Myc+/Strss), a list of 1,069 DEG was retrieved for control, with 712 DEG up-regulated by AMF inoculation, and 357 DEG down-regulated, and a small set of 15 DEG was obtained for water deficit treatments, with 11 DEG up-regulated by the AMF inoculation and 4 DEG down-regulated (**Supplementary Table S3**).

**FIGURE 3:**
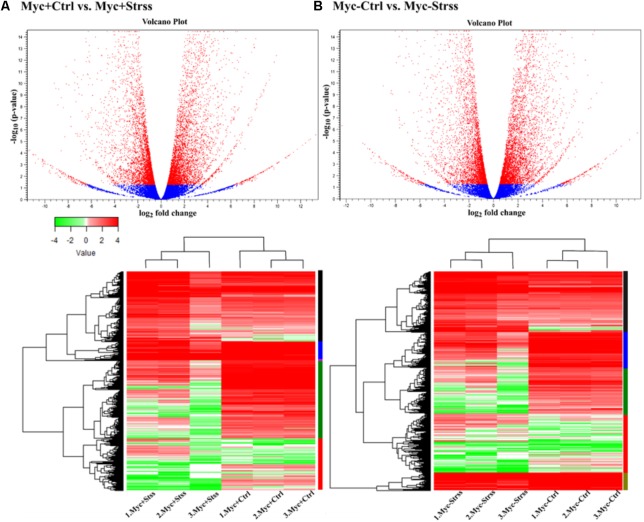
Differential expression analysis of RNA-Seq data. Statistical comparisons: **(A)** AMF-inoculated roots, control vs. water deficit (Myc+/Ctrl vs. Myc+/Strss); **(B)** non-inoculated roots, control vs. water deficit (Myc–/Ctrl vs. Myc–/Strss). At the top, Volcano plots of: **(A)** AMF-inoculated (right) and **(B)** non-inoculated roots (left); transcripts found to significantly up-/down-regulated (*p*-value with FDR < 0.05) are red colored dots. At the bottom, heat maps of all differentially expressed transcripts within biological replicates (*p-value* with FDR < 0.05): **(A)** AMF-inoculated (right) and **(B)** non-inoculated roots (left); colorized bars at the left of heat maps represent cluster of differentially expressed genes (DEGs). Heat maps based on Log_2_ transformed normalized expression data.

Differentially expressed genes that underwent significant changes in the four statistical comparisons can be depicted by the “red” dots in the Volcano plots displayed in **Figure 3**. The range in the expression levels of statistically significant DEGs varies considerably in all sets: from -11.4 to 13.29-fold change in *Myc+*/*Ctrl vs. Myc+/Strss*; from -12.73 to 11.73-fold change in *Myc*-/*Ctrl vs. Myc-/Strss*; from -12.88 to 11.56-fold change in *Myc*-/*Ctrl vs. Myc+*/*Ctrl*; and from -7.97 to 6.4-fold change in *Myc-/Strss vs. Myc+/Strss* (**Figure 3** and **Supplementary Table S3**). DEGs showing the most significant statistical differences (*p-value* with FDR < 0.05) among biological replicates due to the “treatment” factor were selected and heat maps were constructed based on Manhattan distance and complete linkage for both comparisons: *Myc+/Ctrl vs. Myc+/Strss* and *Myc-/Ctrl vs. Myc-/Strss* (**Figure 3**). Two exclusive clusters of differential expressed genes were found, one for each comparison: “pink” bar for AMF-inoculated plants (**Figure 3A**) and “olive” bar for non-inoculated (**Figure 3B**).

**FIGURE 4:**
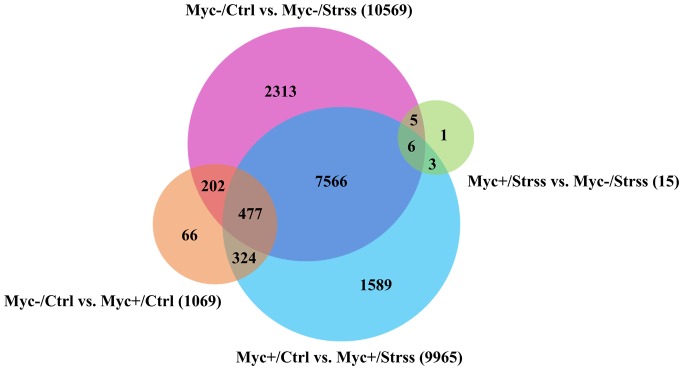
Venn diagram showing relationships among the sets of DEGs retrieved after differential gene expression analysis made between group pairs. Four statistical comparisons include: blue circle: AMF-inoculated roots, control vs. water deficit (Myc+/Ctrl vs. Myc+/Strss); pink-circle: non-inoculated roots, control vs. water deficit (Myc–/Ctrl vs. Myc–/Strss); orange-circle: control roots, non-inoculated vs. AMF-inoculated (Myc–/Ctrl vs. Myc+/Ctrl); green-circle: water deficit treated roots, AMF-inoculated vs. non-inoculated (Myc+/Strss vs. Myc–/Strss). Only DEGs, including transcripts isoforms, with a *p*-value with FDR < 0.05 were considered.

A Venn diagram was generated to further explore the relationships among the different sets of differential gene expression results (**Figure 4**). A total of 8,049 DEG were regulated in both AM- and non-AM, control against water deficit, comparisons. However, some exclusive DEGs were also evidenced for both sets, with non-inoculated plants showing 2,313 exclusive DEG, and AMF-inoculated with 1,589 exclusive DEG. For the list of DEG relative to the *Myc*-/*Ctrl vs. Myc+/Ctrl* set, 66 DEGs are exclusive, with the rest shared between both *control against water deficit* comparisons. From the list of DEG retrieved from the *Myc-/Strss vs. Myc+/Strss* comparison, only one (Phvul.003G105500.1 – *PRMT4b*) is exclusive, with the rest also shared with both *control against water deficit* comparisons. Both *Myc-/Ctrl vs. Myc+/Ctrl* and *Myc-/Strss vs. Myc+/Strss* sets do not share DEG in common. For a list of the exclusive DEG in each set and their related putative functions see **Supplementary Table S4**.

**FIGURE 5:**
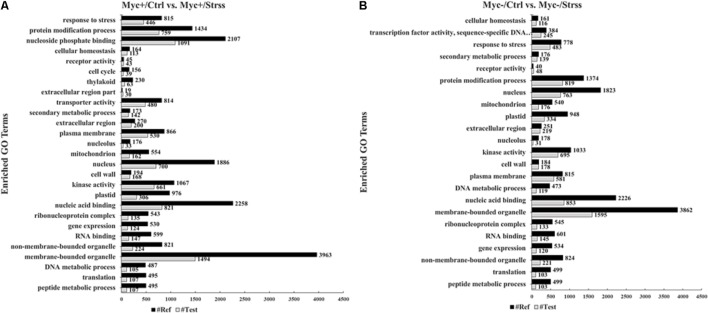
Gene ontology terms over-/underrepresented in both sets of DEGs (**A**, Myc+/Ctrl vs. Myc+/Strss and **B**, Myc-/Ctrl vs. Myc-/Strss) after enrichment analysis performed based on a *Fisher-2 tailed test* at FDR < 0.01. Numbers of DEGs assigned to each GO term are plotted at the right of the bars. Open bars: Test set expressed by the differentially regulated genes; closed bars: Reference set given by total set of *Phaseolus vulgaris* transcripts.

Following gene annotation and functional classification of the lists of DEG retrieved from each statistical comparison, the enrichment analyses of ontology terms were performed based on a *Fisher-2 tailed test* at FDR < 0.01, considering the total set of *P. vulgaris* annotated transcripts as the reference set. For the AMF-inoculated plants, control against water deficit comparison (Myc+/Ctrl vs. Myc+/Strss), a total of 98 ontology terms was differentially represented, with 36 of them over-represented in relation to the reference and 62 down-represented (**Supplementary Table S5**). For the non-inoculated set (Myc-/Ctrl vs. Myc-/Strss), 87 ontology terms showed differential representation, with 27 over-represented terms and 60 down-represented. Individual results of enrichment analysis tests are summarized according to the three major Gene Ontology classes: Biological Process, Molecular Functional, and Cellular Component (**Figure 5**).

The enrichment analyses helped clarifying the dissimilarities found in the DEG’s list composition of both AM- and non-AM, control against water deficit, statistical comparisons displayed in the Venn diagram (**Figure 4**). The inoculation with AMF seemed to interfere with the *cell cycle* (GO:0007049), with this ontology term exclusively under-represented for the *Myc+/Ctrl vs. Myc+/Strss* comparison (**Figure 5A**). Moreover, the molecular functions *nucleoside phosphate binding* (GO:1901265) and *transporter activity* (GO:0005215) were only over-represented for the *Myc+/Ctrl vs. Myc+/Strss* set. Transcripts associated with *thylakoid* (GO:0009579) seemed to be under-represented exclusively in the *Myc+/Ctrl vs. Myc+/Strss* set, while those associated with the constituent parts of *extracellular space* (GO:0005615), including host cell environment outside an intracellular parasite, were over-represented. For the statistical comparison set *Myc-/Ctrl vs. Myc-/Strss*, the enrichment analysis showed an exclusive over-representation of terms associated with the molecular function *transcription factor* (TF) *activity*, *sequence-specific DNA binding* (GO:0003700) (**Figure 5B**).

The over-represented ontology term *response to stress* (GO:0006950), referring to changes in *cellular homeostasis* (GO:0019725) as a result of exogenous factors, was further explored aiming to target transcripts directly associated with host–symbiont interaction and water deficit event response that were exclusively regulated by both inoculation treatments. For the comparison between non-inoculated treatments, control against water deficit (Myc-/Ctrl vs. Myc-/Ctrl), 483 DEGs were assigned to *response to stress*, with 68 of them being exclusive to this condition, and 116 DEGs were associated with *cellular homeostasis*, with 21 of them exclusive. In the AMF-inoculated treatments comparison (Myc+/Ctrl vs. Myc+/Strss), 446 DEGs were assigned to *response to stress*, with 104 exclusives to this comparison, and 113 DEGs were associated with *cellular homeostasis*, with 18 of them exclusive (**Supplementary Table S5**).

This allowed the identification of some interesting common bean genes that are differentially regulated in response to water deficit during AMF inoculation, including those involved in TF activity, carbohydrate biosynthesis, osmoregulation, transport, DNA double-strand break repair, RNA processing, signal transduction, response to oxidative stress, protein ubiquitination, callose deposition, and response to auxin (**Table 1**). Among these genes, ethylene-responsive element binding factors (ERFs), a family of TFs regulated by the gaseous hormone ethylene induced by both biotic and abiotic stresses (Fujimoto et al., 2000), were the most abundant with a total of eight DEGs – including one transcript also containing the APETALA2 (AP2) domain. Also, another important biotic/abiotic stress responsive TFs were highlighted, like the WRKY (five DEGs), the auxin-responsive (six DEGs), and MADS box (one DEG), as well as some genes participating in osmotic adjustment like the aquaporin *PvNIP2;4*, LEA3 protein, Trehalose-phosphate phosphatases (3 DEG), and Na(+) and K(+) transporters (two DEGs).

**Table 1:** Differentially expressed drought-responsive and symbiosis-related genes that were exclusively regulated in AMF-inoculated common bean roots comparing control and treatment (Myc+/Ctrl vs. Myc+/Strss).

Transcript ID^a^	Gene annotation^b^	Putative function^c^	Log_2_ fold-change	*p*-value
Phvul.001G046900.1	Ethylene-Responsive TF (ERF003)	TF^d^	3.75	0.03
Phvul.001G131300.1	AP2-like factor, ANT lineage	TF	1.48	0.02
Phvul.001G196800.3	Nuclear TF Y Subunit A-10	TF	1.07	0.02
Phvul.001G266300.1	Heat Stress TF A-5	TF	1.61	0.04
Phvul.002G016700.1	Ethylene-responsive TF (ERF008)	TF	-1.64	0.05
Phvul.003G116300.1	WRKY48-related	TF	1.81	0.04
Phvul.003G244000.1	Heat stress TF B-1	TF	1.57	0.04
Phvul.003G273900.1	Basic leucine zipper 1-related	TF	-1.64	0.04
Phvul.004G068900.1	Ethylene-responsive TF (LEP)	TF	-2.91	0.01
Phvul.004G163300.1	Heat stress TF C-1	TF	1.67	0.01
Phvul.005G005800.1	WRKY2-related	TF	-1.1	0.03
Phvul.005G080200.1	WRKY DNA-binding domain	TF	2.19	0.03
Phvul.005G105200.1	Ethylene-responsive TF (CRF5)	TF	-2.11	4.02*E*-03
Phvul.005G181800.1	WRKY40-related	TF	-1.14	4.43*E*-04
Phvul.007G065100.1	MADS box protein	TF	3.16	3.82*E*-04
Phvul.007G217800.1	Ethylene-responsive TF (ERF018)	TF	-1.23	6.49*E*-04
Phvul.008G043000.1	WRKY DNA-binding domain	TF	1.19	2.06*E*-03
Phvul.009G123300.1	Ethylene-responsive TF (ERF018)	TF	3.26	0.01
Phvul.009G225000.1	Ethylene-responsive TF (ERF018)	TF	2.63	0.01
Phvul.005G111200.1	Dehydration-responsive element binding (DREB2A)	TF	0.84	5.14*E*-03
Phvul.002G242300.1	Aquaporin (PvNIP1;4)	TA	1.29	6.96*E*-03
Phvul.001G251300.1	Trehalose-phosphate phosphatase	TB	1.92	0.03
Phvul.002G102300.1	α,α-Trehalose-phosphate phosphatase	TB	-1.84	6.15*E*-03
Phvul.003G053000.1	α,α-Trehalose-phosphate phosphatase	TB	-1.49	3.50*E*-07
Phvul.005G002900.3	Far-red impaired responsive 1-like	DR	2.54	1.52*E*-03
Phvul.007G125600.1	DNA-repair protein XRCC4	DR	1.27	2.62*E*-03
Phvul.010G130000.1	Growth-regulating factor 5	TF	-2.19	2.62*E*-03
Phvul.001G156500.1	Auxin-responsive protein	TF	-2.69	2.87*E*-03
Phvul.001G164900.1	Auxin-responsive protein (IAA10)	TF	2.29	1.29*E*-04
Phvul.002G282200.1	Auxin-responsive factor 2	TF	-1.12	0.01
Phvul.005G136400.1	No-apical meristem protein (NAM)	TF	6.82	0.04
Phvul.005G172900.1	Auxin-responsive protein (IAA19)	TF	-1.83	0.05
Phvul.007G189400.2	Auxin-responsive protein (IAA10)	TF	2.13	0.01
Phvul.010G130000.1	Growth-regulating factor 5	TF	-2.19	2.62*E*-03
Phvul.011G080100.1	Auxin-response factor 10	TF	1.01	5.29*E*-03
Phvul.001G207900.1	Late embryogenesis abundant 3 (LEA3)	RS	-1.31	0.04
Phvul.004G155900.1	Protein SAR deficient 1	RS	1.91	0.03
Phvul.008G074100.2	C2-calcium/lipid-binding endonuclease/exonuclease/phosphatase	RS	-2.08	0.01
Phvul.009G201000.2	Adenine nucleotide α-hydrolase-like protein	RS	-1.59	0.02
Phvul.001G011300.1	Peroxidase 9	OR	-1.56	0.03
Phvul.003G078600.1	Peroxidase 42	OR	1.35	0.02
Phvul.009G140700.1	Peroxidase 52	OR	-1.51	4.29*E*-04
Phvul.002G106600.1	RAC-like GTP-binding protein ARAC1-related	ST	1.1	0.03
Phvul.002G263400.1	CAMP-response element binding protein	ST	3.09	4.43*E*-04
Phvul.002G323000.1	LRR-containing protein	ST	-1.05	4.39*E*-03
Phvul.002G324900.1	Histidine kinase	ST	1.35	7.14*E*-03
Phvul.003G015500.2	Histidine kinase	ST	1	0.04
Phvul.004G140800.2	LRR-containing protein	ST	2.27	2.24*E*-03
Phvul.007G167100.1	RhoGAP domain//P-21-Rho-binding domain (PBD)	ST	-1.28	2.00*E*-03
Phvul.008G195000.1	LRR-containing protein	ST	2.88	0.04
Phvul.008G205700.2	CBL-interacting Ser/Thr-protein kinase 3	ST	-1.15	3.70*E*-03
Phvul.010G025400.1	NB-ARC domain (NB-ARC)/TIR domain (TIR)	ST	1.31	3.07*E*-03
Phvul.010G025500.1	NB-ARC domain (NB-ARC)/TIR domain (TIR)	ST	3.32	5.95*E*-05
Phvul.010G029800.1	NB-ARC domain (NB-ARC)/TIR domain (TIR)	ST	2.94	0.04
Phvul.010G132000.1	LRR-containing protein	ST	1.05	0.05
Phvul.010G132200.1	NB-ARC domain (NB-ARC)/(LRR_3)/(TIR_2)	ST	1.17	1.23*E*-04
Phvul.010G136700.1	LRR-containing protein	ST	2.11	3.49*E*-03
Phvul.011G050100.2	Extra-large guanine nucleotide-binding protein 2	ST	1.86	6.62*E*-03
Phvul.002G284700.1	SAUR family protein	RA	-3.09	3.26*E*-03
Phvul.007G227100.1	SAUR family protein	RA	4.26	4.87*E*-06
Phvul.001G115000.1	Na(+)/H(+) exchanger related-1	RA	-1.75	6.90*E*-03
Phvul.001G247300.1	K+/H+-antiporter	RA	-4.3	0.02
Phvul.003G089800.1	Na(+)/H(+) exchanger related-5	RA	-9.61	4.29*E*-03
Phvul.002G331800.1	WDSAM1 protein	PU	-2.66	7.11*E*-03
Phvul.007G072300.2	U-box domain-containing protein 32	PU	1.22	9.85*E*-03
Phvul.009G119200.1	Inhibitor of apoptosis	PU	2.36	0.02
Phvul.010G099400.1	U-box domain-containing protein 33	PU	1.99	0.01
Phvul.009G068400.1	Mortality 4-like protein 1	CM	1.16	6.33*E*-03
Phvul.003G007700.2	Histone–Lys *N*-methyltransferase, H3 Lys-9-specific SUVH7	HM	-1.12	0.05
Phvul.001G134900.1	Zn-finger AN1 and C2H2 domain-containing stress-associated protein 11	MB	-1.53	0.03
Phvul.003G243300.1	MAPKKK19	ST	-3.27	1.61*E*-08
Phvul.004G174500.2	MAPKK6	ST	2.69	0.03
Phvul.003G275100.1	U2-associated protein SR140	RP	-10.81	7.60*E*-04
Phvul.005G036700.1	tRNA (cytidine(34)-2’-*O*)-methyltransferase	RP	1.27	0.02
Phvul.006G127100.2	endoribonuclease Dicer (DICER1, DCR1)	RP	-1.33	0.02


*^*a*^According to the *Phaseolus vulgaris* reference genome v.11.0 available at Phytozome. ^*b*^Confirmed by the PhytoMine tool using *P. vulgaris* annotation data (Phytozome v11). ^*c*^Gene Ontology functional classification data for *P. vulgaris* (Phytozome v11). ^*d*^GO terms: TF, transcription factor; TA, transport activity; SB, sucrose biosynthesis; TB, trehalose biosynthesis; GD, 1,3-beta-D-glucan biosynthesis; RS, response to stress; OR, oxidative response; ST, signal transduction; DR, double-strand break repair; RA, solute: proton antiporter activity; PU, protein ubiquitination; CM, chromosome modification; HM, histone modification; MB, metal ion binding; RP, RNA processing.*

**FIGURE 6:**
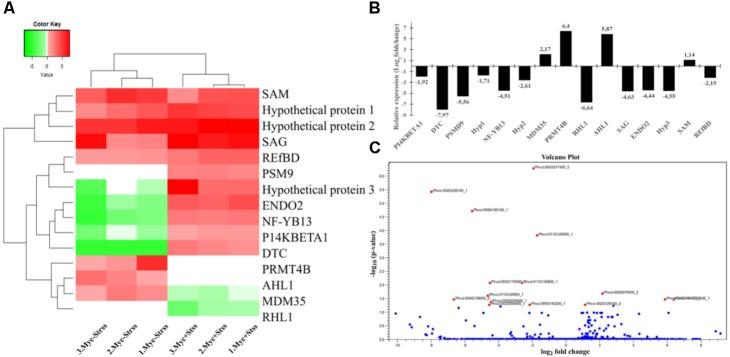
RNA-Seq differential gene expression analysis comparinganalysis comparing water deficit treatments non-inoculated against AMF-inoculated common bean roots (Myc–/Strss vs. Myc–/Strss). **(A)** Heat maps of all differentially expressed transcripts within biological replicates (*p-value* with FDR < 0.05). Heat maps based on individual Log_2_ transformed normalized expression data. **(B)** Log_2_ transformed relative expression data comparing the means of normalized counts retrieved for each DEG in both conditions. **(C)** Volcano plot; transcripts found to significantly up-/down-regulated (*p-value* with FDR < 0.05) are red colored dots.

The list of DEG retrieved from the statistical comparison between control treatments (Myc-/Ctrl vs. Myc+/Ctrl) revealed an exclusive enrichment for the biological process *response to biotic stimulus* (GO:0009607), and for transcripts associated with *lysosome* (GO:0005764) (**Supplementary Table S5**). For water deficit event treatments (Myc-/Strss vs. Myc+/Strss), an enrichment analysis of ontology terms was not possible due to the small amount of DEG retrieved. However, gene annotation allowed the assignment of putative function to 12, of the 15 DEG: one RNA-binding protein (*REfBD*) (Phvul.005G142200.1) (Glisovic et al., 2008); one single-strand-specific nuclease, *ENDO 2* (Phvul.003G030500.1) (Desai and Shankar, 2003); one *S*-adenosyl-L-methionine-dependent methyltransferase (*SAM*) (Phvul.002G129100.2) (Kozbial and Mushegian, 2005); the TF *AHL1* (Phvul.008G076100.2), usually associated with mitosis regulation (Fujimoto et al., 2004); two transcripts involved in mitochondrial metabolism, *DTC* (Phvul.002G238100.1) (Dolce et al., 2014) and *MDM35* (Phvul.009G079400.2) (Miliara et al., 2015); one *SAG* gene (Phvul.011G120900.1), probably associated with the transference of senescence signals during drought (Wehner et al., 2016); and four transcripts associated with plant development, like the flowering time regulator *PRMT4B* (Phvul.003G105500.1) (Niu et al., 2008), and those related to root hair formation *RHL1* (Phvul.004G139000.2) (Schneider et al., 1998), elongation *PI4KBETA1* (Phvul.002G271500.2), and the TF *NF-YB13* (Phvul.003G179300.2) implicated in primary root elongation and drought-stress response (Quach et al., 2015; **Figure 6** and **Supplementary Table S3**).

### Temporal Shift of Aquaporin Gene Expression in Roots and Leaves

Arbuscular mycorrhization has been implicated in the maintenance of common beans RWC, transpiration, and root hydraulic conductivity (*L*p_R_) through a fine-tune regulation in the expression levels of genes encoding aquaporins (Aroca et al., 2006), making this gene family perfect candidates for our time-course relative gene expression analysis by RT-qPCR.

Considering the 41 *P. vulgaris* aquaporin-related transcripts (Ariani and Gepts, 2015), differential gene expression analysis of RNA-Seq data showed that 18 transcripts were up-regulated, and four down-regulated, in AMF-inoculated roots under regular irrigation control conditions in relation to the water deficit event (Myc+/Ctrl vs. Myc+/Strss), with the gene *PvNIP1;4* (Phvul.002G242300) exclusively regulated (1.29-fold change) under mycorrhizal colonization (**Supplementary Table S6**). For non-inoculated treatments, 22 transcripts were up-regulated and 5 were down-regulated, in control plants in relation to water deficit, with six of them exclusively regulated: *PvPIP2;1* (Phvul.004G082600) (5.81-fold change), *PvTIP2;1* (Phvul.005G170300) (4.34-fold change), *PvSIP1;1* (Phvul.001G097000) (0.67-fold change), *PvSIP1;3* (Phvul.011G102700) (3.68-fold change), *PvSIP2;1* (Phvul.001G108800) (0.59-fold change), and *PvXIP1;2* (Phvul.011G025800) (-3.92-fold change). Hence, genes belonging to the aquaporin subfamily *small-intrinsic proteins* (SIPs) were only found differentially regulated under non-inoculated conditions.

Nine aquaporin-related transcripts were selected for the RT-qPCR analysis based on their previous reported relevance for drought-stress response in common bean’s BAT477 genotype (Recchia et al., 2013). Total RNA was obtained from both roots and leaves harvested at four periods, 24, 48, 72, and 96 h of the water deficit event treatment. Three technical replicates were considered for each of the biological triplicates from the four initial treatments. Relative expression analysis between treatments were performed taking *IDE* and *UBQ* genes as internal normalizers for leaf samples and *IDE* and *Skip16* genes for root samples (Borges et al., 2012). Three statistical comparisons were performed: *Myc+/Strss vs. Myc+/Ctrl*, *Myc-/Strss vs. Myc-/Ctrl* and *Myc+/Strss vs. Myc-/Strss*.

**FIGURE 7:**
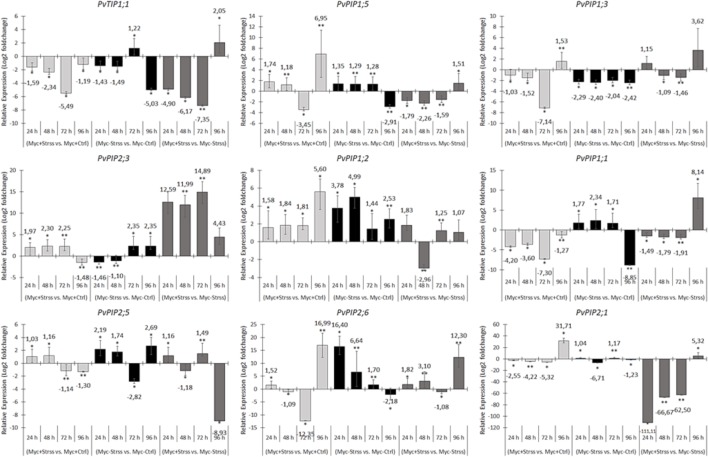
RT-qPCR-based relative gene expression analysis of aquaporin-related transcripts in common bean roots harvested at 24, 48, 72, and 96 h of treatment. For each transcript, three relative comparisons were made between treatments: *Myc+/Stss vs. Myc+/Ctrl* (AM-inoculated, water deficit vs. control), *Myc*–*/Strss vs. Myc*–*/Ctrl* (non-inoculated, water deficit vs. control) and, *Myc+/Strss vs. Myc*–*/Strss* (water deficit, AM-inoculated vs. non-inoculated). Relative expression levels were log_2_ transformed and the average fold changes in expression ratio for each gene is highlighted. Statistical hypothesis testing based on *p-values* were obtained by a pairwise reallocation randomization test (bootstrap = 2,000 permutations). Bars represent the standard error of the mean factor of relative expression results comparing sample and control groups.

**FIGURE 8:**
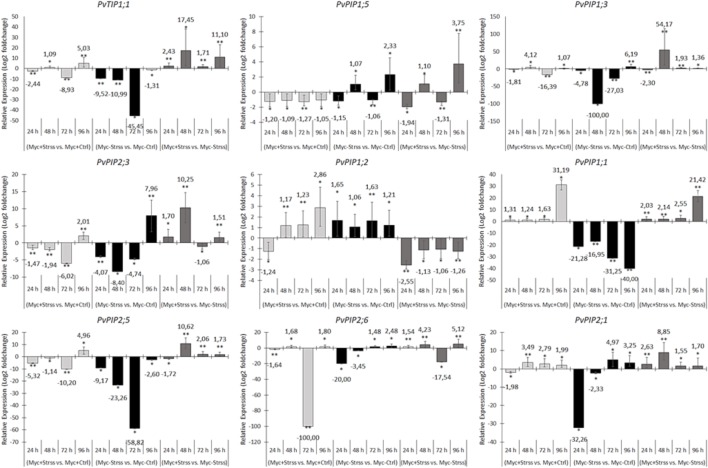
RT-qPCR-based relative gene expression analysis of aquaporin-related transcripts in common bean leaves harvested at 24, 48, 72, and 96 h of treatment. For each transcript, three relative comparisons were made between treatments: *Myc+/Stss vs. Myc+/Ctrl* (AM-inoculated, water deficit vs. control), *Myc*–*/Strss vs. Myc*–*/Ctrl* (non-inoculated, water deficit vs. control) and, *Myc+/Strss vs. Myc*–*/Strss* (water deficit, AM-inoculated vs. non-inoculated). Relative expression levels were log_2_ transformed and the average fold changes in expression ratio for each gene is highlighted. Statistical hypothesis testing based on *p-values* was obtained by a pairwise reallocation randomization test (bootstrap = 2,000 permutations). Bars represent the standard error of the mean factor of relative expression results comparing sample and control groups.

The overall performance of up- and down-regulation of aquaporin transcripts in both organs and inoculation treatments was very random through the 96 h of water deficit treatment (**Figures 7**, **8**). Thus, grouping the differential expression results into clusters sharing similar profiles of up-/down-regulation was not possible for the statistical comparisons *Myc+/Strss vs. Myc+/Ctrl* and *Myc-/Strss vs. Myc-/Ctrl*. Statistical significant differences (*p*-value < 0.05) in the expression regulation of these transcripts in roots and leaves were found in the four periods tested. The only exceptions are the gene *PvPIP1;2* that in roots was up-regulated by water deficit in relation to control in both inoculation conditions, and *PvPIP2;3* that in leaves was down-regulated for the initial 48 h by water deficit when compared to the control, with an up-regulation at 96 h, also for both inoculation systems (**Figures 7**, **8**).

Relative gene expression data of root samples comparing both water deficit treatments (Myc+/Strss vs. Myc-/Strss) revealed no significant statistical changes at 24 and 96 h (**Figure 7**). The majority of aquaporin transcripts tested showed significant down-regulation in water deficit event AMF roots in relation to non-AM at both 48 and 72 h: *PvTIP1;1*, *PvPIP1;5*, *PvPIP1;3*, *PvPIP1;1*, and *PvPIP2;1*; one was up-regulated at both periods, *PvPIP2;3*; two were down-regulated at 48 h and down-regulated at 72 h: *PvPIP1;2* and *PvPIP2;5*; and *PvPIP2;6* was up-regulated at 48 h and down-regulated at 72 h.

Regarding leaf samples of water deficit event treated plants, the expression levels of *PvPIP1;2* were lower in mycorrhizal plants, in relation to non-inoculation treatment, for the whole period of water deficit treatment (**Figure 8**). Three other genes, *PvTIP1;1*, *PvPIP1;1*, and *PvPIP2;1*, showed superior levels of expression in Myc+/Strss leaves in relation to Myc-/Strss for the whole period, with peaks of up-regulation of 17.45-fold at 48 h for *PvTIP1;1*, 21.42-fold at 96 h for *PvPIP1;1* and 8.25-fold at 48 h for *PvPIP2;1*. Four other genes, *PvPIP1;3*, *PvPIP2;5*, *PvPIP2;3*, and *PvPIP2;6*, are up-regulated in Myc+/Strss samples in relation to Myc-/Strss for all periods, except for a down-regulation at the initial 24 h for *PvPIP1;3* and *PvPIP2;5*, and at 76 h for *PvPIP2;3* and *PvPIP2;6*. For *PvPIP1;3*, *PvPIP2;5*, and *PvPIP2;3*, this down-regulation is related to a lower repression rate of these genes in leaves of non-inoculated plants during the water deficit event, and a pronounced down-regulation of 100-fold for *PvPIP2;6* in Myc+/Strss samples after 72 h of treatment.

### Tissue-Specific Gene Expression Analysis

The establishment of AMF specialized colonization structures – termed “arbuscules” – in root cortical cells are accompanied by an intense modification in host’s transcriptome, both at local and systemic levels (Harrison, 2005; Balestrini and Lanfranco, 2006). To better characterize this influence in our model of study, the microscopy-based LCM technique was applied in combination with relative RT-qPCR analysis. A set of 23 transcripts were chosen among the main functional classes that were enriched by our treatments. Three cell populations were collected: ctx+ – root cortical cells containing arbuscules and/or hyphal structures derived from Myc+/Strss treatments; ctx- – root cortical cells not containing arbuscules derived from Myc+/Strss treatments; and ctrl – root cortical cells derived from Myc-/Strss treatments (**Figures 1D–I**).

The paraffin embedding and tissue staining protocols showed to be adequate to preserve internal root and AM colonization structures and was helpful to secure RNA molecules integrity. The complete protocol, from tissue sectioning, through RNA extraction and cDNA synthesis, was always performed in a row, with the newly obtained cDNA samples kept at -20°C to prevent contamination. For the relative RT-qPCR analysis, three biological replicates, containing technical triplicates each, were considered.

The combination of paraffin embedding and the selected staining protocol, however, affected the resolution of the histological sections. This did not compromise the proper identification of internal root and AM colonization structures but opened the possibility for a detrimental effect of such a prolonged water deficit treatment (96 h) over arbuscules integrity. Therefore, histological sections retrieved from the same pools were embedded in historesin and stained following the same protocols, offering superior resolution and confirming an extensive AM colonization net through root cortical cells that are interconnected by hyphal structures (**Figures 1A,B**) and arbuscules at alternating stages of development (**Figure 1C**), thus revealing that colonization is an ongoing process even during environmental stressful conditions.

RT-qPCR amplification products were visualized in a 1.2% agarose gel (**Supplementary Figure S2a**). From the selected internal normalizers, *skip16* was the most stable in all samples analyzed (**Supplementary Figure S2b**). AML1 and AML2 amplification attested the quality of microdissected samples since no amplification products were detected on both ctx- and ctrl samples (**Supplementary Figure S2c**). Products from two transcripts, *GH3* (Phvul.004G077000) and *PvPIP2;3* (Phvul.007G094600), were only amplified in ctx+ samples. Five other transcripts, *bhlh95* (Phvul.003G048000), *CKX* (Phvul.009G081800), *bZIP* (Phvul.010G018200), *KHX* (Phvul.001G051900), and *ABHD* (Phvul.006G155700), were amplified only in ctx+ and ctrl cells. Amplification products were obtained from the other 16 transcripts for the three cell types analyzed. No-template control confirmed the absence of genomic-DNA contamination and the formation of primer artifacts.

**FIGURE 9:**
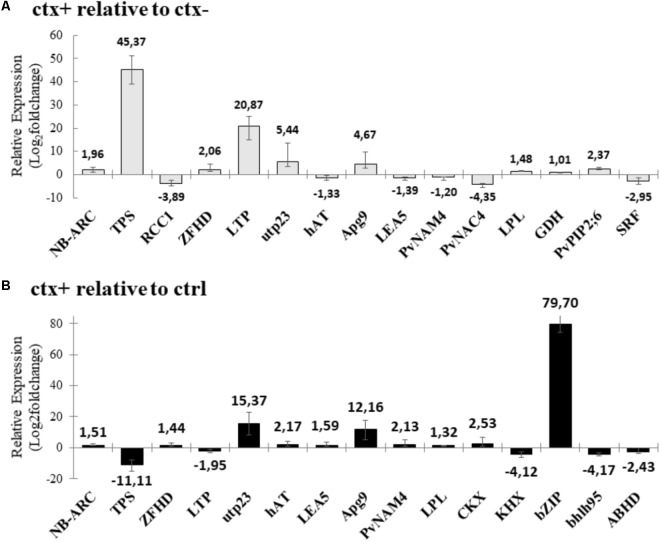
Relative gene expression data analysis using real-time quantitative PCR. **(A)** Water deficit treated root-cortical cells containing arbuscules (ctx+) relative to water deficit treated non-arbusculated AM-root cortical cells (ctx–); **(B)** water deficit treated root cortical cells containing arbuscules (ctx+) relative to water deficit treated root-cortical cells retrieved from non-inoculated plants (ctrl). Relative expression levels were log2 transformed and the average fold changes in expression ratio for each gene is highlighted. Only genes with significant changes (*p* < 0.05) are shown. *p*-values were obtained by a pairwise reallocation randomization test (bootstrap = 2,000 permutations). Bars represent the standard error of the mean factor of relative expression results comparing sample and control groups.

Two relative gene expression analyses were performed: *ctx+ relative to ctx-* and *ctx+ relative to ctrl* (**Figure 9**); both were applied to identify differences in transcription regulation due to the presence of arbuscules. Among those genes that underwent significant changes in expression in arbusculated root cortical cells (ctx+) relative to their neighboring non-arbusculated root-cortical cells (ctx-), nine were up-regulated in ctx+ samples: *NB-ARC-LRR* (Phvul.001G132700) (1.96-fold), *TPS* (Phvul.011G107200) (45.57-fold), *ZFHD* (Phvul.009G037900) (2.06-fold), *LTP* (Phvul.008G198300) (20.87-fold), *utp23* (Phvul.005G025400) (5.44-fold), *Apg9* (Phvul.007G194300) (4.67-fold), *LPL* (Phvul.008G198300) (1.48-fold), *GDH* (1.01-fold) (Phvul.002G122600), and *PvPIP2;6* (Phvul.011G079300) (2.37-fold); and six were down-regulated: *RCC1* (Phvul.008G230700) (-3.89-fold), *hAT* (Phvul.001G020700) (-1.33-fold), *LEA5* (GI| 17325561|) (-1.40-fold), *PvNAM4* (Phvul.009G038400) (-1.25-fold), *PvNAC4* (Phvul.009G038400) (-4.55-fold), and *SRF* (Phvul.008G027900) (-2.95-fold) (**Figure 9A**).

Relative gene expression analysis between arbusculated cells from AMF-inoculated roots (ctx+) relative to cortical cells from non-inoculated roots (ctrl) showed that 10 transcripts were up-regulated: *NB-ARC-LRR* (1.51-fold), *ZFHD* (1.44-fold), *utp23* (15.37-fold), *hAT* (2.17-fold), *LEA5* (1.59-fold), *Apg9* (12.16-fold), *PvNAM4* (2.13-fold), *LPL* (1.32-fold), *CKX* (Phvul.009G081800) (2.53-fold), and *bZIP* (Phvul. 010G018200) (79.7-fold); and five were down-regulated: *TPS* (-11.11-fold), *LTP* (-1.95-fold), *KHX* (Phvul.011G019400) (-4.12-fold), *bhlh95* (Phvul.003G48000) (-4.17-fold), and *ABHD* (Phvul.006G155700) (-2.43-fold) (**Figure 9B**).

Some of the selected genes were amplified in the three cell types, but relative expression did not reveal and significant statistical changes between the conditions tested. This was the case for *PvNAM28* (Phvul.005G047300) in the *ctx+ vs. ctx-* analysis. For the *ctx+ vs. ctrl*, in addition to *PvNAM28*, five other transcripts could be pointed: *RCC1*, *LEA5*, *PvNAC4*, *GDH*, and *PvPIP2;5*. The *SRF* gene was only amplified in AMF-inoculated roots, with a down-regulation in *ctx+ vs. ctx-* (**Figure 9A**). For the transcripts with significant differential regulation exclusively for the *ctx+ vs. ctrl* comparison, two were up-regulated in ctx+ samples, *CKX* and *bZIP*, and three were down-regulated, *KHX*, *bhlh95*, and *ABDH* (**Figure 9B**). Genes *GH3* and *PvPIP2;3* were only amplified in ctx+ samples, therefore not producing relative gene expression data.

## Discussion

The examination of the AMF colonization influence over the gene expression profile of plants has been conducted for a variety of species (Güimil et al., 2005; Zouari et al., 2014; Handa et al., 2015; Vangelisti et al., 2018). In all studies, AMF colonization seemed to affect a diverse range of metabolic pathways during plant development, including primary and secondary metabolisms, ion transport, signal transduction, and transcriptional regulation, indicating a strong dependency by the hosts to this kind of symbiosis.

In our study, AMF colonization did not produce any distinguishable morphologic alterations in plants during the water deficit regime. Although a slightly increase in RLD and leaf dry matter (LDM) was observed for the AMF-inoculated well-watered plants (Myc+/Ctrl), no statistical significant changes were observed for the AMF-inoculated water deficit treated plants (Myc+/Strss) when compared with non-inoculated treatments, despite root AMF colonization being 1.33 times higher in average for the Myc+/Strss treatment (74.44%) than for Myc+/Ctrl (55.74%). Although an increase in AM-root colonization during water scarcity regimes is not expected, especially due to a reduced availability of carbon from the host (Subramanian and Charest, 1995; Zhang et al., 2014; Gong et al., 2015), studies involving potted plants with relatively short water deficit treatments demonstrated that the duration of drought has not necessarily appeared to favor or discourage colonization, with both observations being reported (Augé, 2001).

Similarly, photosynthetic variables were mostly altered due to the water deficit treatment, but no significant differences, in general, were detected between inoculated and non-inoculated plants under stress. Mycorrhizal influences on tissue hydration and foliar gas exchange are often subtle, transient, and probably circumstance and symbiont specific. Although several reports have indicated an increase in photosynthetic rates of plants during the symbiosis (Augé, 2001; Bárzana et al., 2012; Rapparini and Peñuelas, 2014), many investigators found no differences between AM and non-AM plants, especially regarding stomatal conductance and transpiration (Augé, 2001).

At the molecular level, however, from the 31,638 annotated transcripts in the *P. vulgaris* genome (Schmutz et al., 2014), 9,965 were differentially regulated in AM-inoculated water deficit treated plants in relation to the respective control (Myc+/Ctrl vs. Myc+/Strss), and in non-inoculated conditions this number was even higher, with 10,569 DEG. From these, 1,596 DEGs were exclusively regulated in AM roots and 2,313 DEG in non-AM roots (**Figure 4** and **Supplementary Tables S3**, **S4**). Enrichment analysis revealed that 559 transcripts could be directly assigned to stress response (125 DEG exclusive) and maintenance of cellular water homeostasis in AM-inoculated roots, while for the non-inoculation conditions rates are higher, with 599 DEGs (86 DEG exclusive) (**Supplementary Table S5**). Thus, during arbuscular mycorrhization, common bean plants showed alternative mechanisms to cope with the water deficit event (**Table 1**).

Arbuscular mycorrhizal fungi-inoculated and non-inoculated gene expression profiles were statistically compared to check whether those shifts in plants metabolism could interfere with common beans response to the water deficit event. Although a considerable number of transcripts were differentially regulated between the well-watered control treatments (Myc-/Ctrl vs. Myc+/Ctrl), only 15 statistically significant DEGs were retrieved from the *Myc-/Strss vs. Myc+/Strss* comparison (**Supplementary Table S3**). Thus, those differences highlighted in both control versus water deficit differential analysis might be more related to the dissimilarities guarded between both control treatments (Myc+/Ctrl and Myc-/Ctrl) considered as basis for both statistical comparisons.

Nevertheless, the up-regulation in Myc+/Strss samples, in relation to Myc-/Strss, of two genes, *DTC* and *MDM35*, both associated with mitochondrial metabolism, the exclusive down-regulation of the flowering-time regulator *PRMT4b*, and the up-regulation of genes related to root hair formation *RHL1*, and elongation, *PI4KBETA1* and *NF-YB13*, the latter also involved in water deficit event response, indicate that changes in the plant metabolism and development, especially those associated with root growth and architecture, might occur during the symbiosis, and could potentially affect common beans capacity of responding to a water deficit event. Up-regulation of these genes could also be associated with the discreet increase in RLD and RDM of mycorrhizal roots in relation to non-inoculated samples (**Figure 2**).

Nearly all plant hormones are employed by plants to regulate the symbiosis with AMF (Cosme et al., 2016). Among these signaling molecules, abscisic acid (ABA), a sesquiterpenoid hormone derived from carotenoids, functions at multiple levels to regulate AM symbiosis (Martín-Rodríguez et al., 2016). In many plant species it has been observed that the concentration of ABA rises during the establishment of mycorrhization (Herrera-Medina et al., 2007; Ludwig-Müller, 2010), with evidences of an impact over the proper formation of arbuscules and a sustained colonization of plant roots (Herrera-Medina et al., 2007). During drought, the increased concentration of ABA in leaves promotes the closure of stomata to minimize transpirational water loss. It also mitigates stress damage through the activation of many stress-responsive genes that encode enzymes for the biosynthesis of compatible osmolytes and LEA-like proteins, increasing plant tolerance (Herrera-Medina et al., 2007). Root colonization favors the production of essential isoprenoids, such as carotenoids, therefore, with a direct impact in the source of important growth regulators such as ABA. Thus, it was proposed that a higher demand of carotenoid-derived compounds and pigments is expected in AM plants, especially under stress conditions where these isoprenoid compounds might play a role in plant protection and defense (Rapparini and Peñuelas, 2014).

The relevance of ABA in the response of plants to a water deficit event, especially under AMF colonization, is highlighted in our study by the differential regulation of target genes directly associated with the ABA biosynthesis pathway in both statistical comparisons Myc+/Ctrl vs. Myc+/Strss and Myc-/Ctrl vs. Myc-/Strss (**Supplementary Table S3**). The expression of a zeatin peroxidase *AtZEP* gene homolog (Phvul.002G18700.1) was induced by water deficit in both inoculated (3.51-fold) and non-inoculated treatments (3.19-fold). The 9-*cys-*eppoxycarotenoid dioxygenase genes (*NCED*) were also differentially regulated; an *AtNCED9* homolog (Phvul.005G051600.1) underwent a 4.54-fold change in water deficit treated AM-inoculated plants and a 5.12-fold-change in non-inoculated plants, while the *AtNCED1* homolog (Phvul.009G109100.1) was repressed in water deficit treated plants, with a -8-fold change in AM-inoculated plants and a -10.03-fold change in non-AM plants. A short-chain alcohol dehydrogenase/reductase *ATSDR1* gene homolog (Phvul.011G097200.1) also underwent repression under water deficit conditions in both inoculation treatments, with a -3.51-fold change for AM plants and a -4.71-fold change for non-AM plants. Thus, the differential regulation of these pivotal genes helps not only to validate the efficacy of the water deficit assay, but also highlights that, considering both statistical comparisons, Myc+/Ctrl vs. Myc+/Strss and Myc-/Ctrl vs. Myc-/Strss, the lack of variation between the levels of induction/repression of those genes helps to corroborate the absence of significant statistical differences displayed by the gas exchange measurements considering Myc+/Strss and Myc-/Strss plants (**Figures 2E–H**).

Moreover, two *ABF2* homolog genes (Phvul.009G065500 and Phvul003G291800), both coding for an ABRE-BINDING protein factor (AREB/ABF), were up-regulated with statistical significance only in water deficit treated AM-inoculated plants over the respective control (Myc+/Ctrl vs. Myc+/Strss); with a 2.01-fold change for Phvul.009G065500 and a 1.35-fold change for Phvul003G291800. Both *in vitro* and *in vivo* data indicate that the three AREB/ABF members (*ABF1*, *ABF2*, and *ABF3*) could recognize the ABRE sequence in the *DREB2A* gene promoter in *Arabidopsis* plants and activate it in response to osmotic stress conditions under which activation levels were reduced (Kim et al., 2018). In fact, a *DREB2a* gene homolog (Phvul.005G111200.1) was exclusively regulated in AM-inoculated plants (0.84-fold-change) (**Table 1**). Together, these results point toward alternative mechanisms by which mycorrhizal colonization might influence water deficit response in plants.

As evidences for a mycorrhizal-based water deficit event response in common beans were offered, we decided to test whether a prolonged water deficit treatment (96 h) could impose limiting conditions for AM development, therefore potentially affecting its influence over plant’s metabolism. A RT-qPCR-based relative differential gene expression analysis was performed considering whole roots and leaves, harvested at 24, 48, 72, and 96 h of water deficit treatment – from both AMF-inoculated and non-inoculated, control and stressed plants – aiming to further investigate the influence of mycorrhization over plant’s expression of drought-responsive genes in earlier periods of water deprivation. For this analysis, candidate genes encoding putative aquaporins were considered as markers for water deficit stress response.

Aquaporins provide a low resistance pathway for the movement of water across a membrane, and since aquaporins can be gated, this provides greater control for the movement of water along plant tissues (Bárzana et al., 2014). PIP and TIP isoforms have been recognized as central pathways for transcellular and intracellular water transport (Maurel et al., 2008). Thus, it seems likely that mycorrhizal symbiosis causes significant changes in aquaporin activity of host plants (Porcel et al., 2006; Aroca et al., 2007; Uehlein et al., 2007) and some of the plant aquaporins might be important for these mycorrhizal responses.

In fact, RNA-Seq differential gene expression analysis data collected from both inoculation conditions (Myc+/Ctrl vs. Myc+/Strss and Myc-/Ctrl vs. Myc-/Strss) were further investigated and significant differences in the regulation of the 41 *P. vulgaris* aquaporin-related transcripts were observed; including the existence of certain genes being exclusively regulated in each inoculation condition. In general, a statistical significant down-regulation was observed in most of those transcripts accessed in both inoculation treatments when exposed to the water deficit events. Similar results were found by Bárzana et al. (2014) when analyzing AMF-inoculated maize plants exposed to a severe drought regime, with the down-regulation associated to a mechanism of reducing osmotic root hydraulic conductance in order to prevent excessive water loss to the soil.

Regarding the time-course RT-qPCR analysis, aquaporin genes showed distinct patterns of up-/down-regulation among the different inoculation treatments, organs and time-periods analyzed, therefore not allowing the grouping of those transcripts per common features. This might be a reflect of the redundant nature of the MIP family (*membrane intrinsic protein*) with members that show specific spatial and/or temporal expression patterns, limited to certain organs or cell types, or developmental stages (Javot and Maurel, 2002; Alexandersson et al., 2010). Moreover, Valot et al. (2005) and Aroca et al. (2006) found that several plasma membrane (PM) proteins were differently regulated by inoculation with *G. intraradices*, with some of them being down- or up-regulated. Also, a compensatory mechanism that balances the down-regulation of host’s aquaporins with the heightened activity of AMF aquaporins to maintain high root hydraulic conductance in AM roots has been proposed (Aroca et al., 2009).

Comparing the differential expression profiles of aquaporin genes obtained for water deficit treated roots (Myc+/Strss vs. Myc-/Strss), statistical significant results (*p*-value < 0.05) were retrieved only for intermediate time-periods (48 and 72 h), thus validating initial RNA-Seq results and pointing toward a scenario were AMF inoculation might be more relevant for the regulation of root hydraulic properties at initial stages of water deficit periods. Additionally, although the transcriptional control of aquaporins in water deficit treated leaves is often reported as more complex and a tendency to overall aquaporin gene down-regulation has been reported (Maurel et al., 2008), in our analyses, up-regulation of aquaporin genes in leaves was detected for all the periods analyzed, including *PvPIP1;1*, up-regulated by water deficit in AMF-inoculated plants during the whole period; the same happening for *PvPIP1;2* in non-inoculated plants (**Figure 8**).

Leaves harvested from water deficit event treated samples showed significant up-regulation of aquaporin transcripts (excepting *PvPIP1;2*) in AMF-inoculated plants, in relation to non-inoculated samples, after 96 h (**Figure 8**). Leaf aquaporins, especially those localized in stomata guard cells, play a pivotal role in transpiration (Maurel et al., 2008), and many studies have shown an enhancement in the rates of gas exchange parameters (stomatal conductance, transpiration, and photosynthetic rates) in mycorrhizal plants under water limited conditions (Rapparini and Peñuelas, 2014). The lack of statistically significant differences in the expression levels of drought-related genes between AMF-inoculated and non-inoculated roots could be part of a differentiated response mediated by AMF that prioritizes the regulation of the water relations in aerial parts of the plant in detriment of the root system. Therefore, as the water deficit event increases, mycorrhizal colonization would reinforce previous phenotypic observations made for common bean drought-tolerant lines involving a fine control of stomatal conductance, and an efficient CO_2_ diffusion and fixation, that leads to an effective water use that favors seed filling rather than the maintenance of leaf turgor and growth (Rosales et al., 2012).

Transcripts of a number of genes expressed in mycorrhizal roots occur in cortical cells containing the arbuscules (Balestrini and Lanfranco, 2006), and an AM-specific signal may be responsible for the activation of these genes (Harrison, 2005). Some other genes might be expressed in cortical cells in the vicinity of colonized cells, or elsewhere in the plant, like in leaves, suggesting the presence of a second mobile signal acting in the colonized region of the root (Balestrini and Lanfranco, 2006). Thus, a lot have been questioned about in which extent whole-organs transcriptomic analysis wouldn’t mask the real changes in the transcription levels of genes that are relevant for mycorrhizal symbiosis and are restricted to specific tissues.

The LCM technique was applied and provided successful isolation of root cortical tissues in water deficit treated samples of both AM- and non-AM common bean plants. A set of 23 transcripts were selected among the statistical comparisons of differential gene expression analyses. The selection took into consideration the potential role of these genes in coordinating drought-tolerance responses in common beans under the influence of mycorrhizal colonization and other important ontology terms that were significantly impacted by our treatments, like host–symbiont interaction, plant cellular modifications to harbor arbuscules, transcriptional and post-transcriptional regulation, and genes associated with the control of plant hormonal levels.

Arbuscules are surrounded in cortical cells by a plant-derived pathogen-associated molecule (PAM) that is continuous to the PM of plants and excludes the fungus from the cytoplasm (Parniske, 2008). Thus, the lipid composition of PAM might differ from that of PM (Pumplin and Harrison, 2009). The *LPL* transcript, a lipoprotein lipase (*LPL*) gene, was up-regulated in arbusculated cortical cells (ctx+), both in relation to ctx- (1.48-fold) and to ctrl (1.32-fold). These results are in agreement with the up-regulation of a class 3 lipase (mtr.13800.1s1) in root cortical cells of *Medicago truncatula* inoculated with *G. intraradices* (Gaude et al., 2012).

Pathogen-associated molecule also holds a unique protein composition, including exclusively expressed phosphate transporters (Harrison et al., 2002), H^+^-ATPases (Krajinski et al., 2000), and major intrinsic proteins (MIPs) (Uehlein et al., 2007). Two putative common bean aquaporin transcripts, *PvPIP2;3* and *PvPIP2;6*, were evaluated in our LCM/RT-qPCR analysis. *PvPIP2;3* was exclusively amplified in arbusculated cells (ctx+) (**Supplementary Figure S2**). Since the time-based RT-qPCR analyses showed a down-regulation of this gene in the whole root organ after 96 h of water deficit treatment in AMF-inoculated plants this could be an evidence of potential masking effects of whole transcriptomics analyses in relevant genes regulated by the symbiosis. Moreover, the 16.99-fold up-regulation of *PvPIP2;6* in 96 h water deficit treated AMF-inoculated roots in relation to the respective control and the down-regulation (-2.18-fold) at the same period for non-inoculation conditions (**Figure 7**) could offer, in combination with LCM/RT-qPCR data, the basis for an extra scenario in water deficit tolerance mediated by AMF where transcripts associated with water transport and osmoregulation are induced in arbusculated cortical cells and in neighboring cells through a signalization cascade.

The osmoregulation of cytosolic water homeostasis, a vital process required to sustain cell turgor and support expansion growth of the root system, is achieved by absorption, transport, and compartmentalization of water and solutes, being the intracellular K^+^ levels a prerequisite for the optimal plant metabolic machinery (Shabala and Pottosin, 2014). Liu et al. (2006) indicated that during dehydration, transmembrane channels for water and K^+^ are down-regulated in a coordinated manner, reducing membrane water permeability and promoting cellular water conservation. The *KHX* transcript, coding for a K+/H+ antiporter, was down-regulated in ctx+ cells in relation to ctrl (**Figure 9B**). Since *KHX* was not amplified in ctx- samples, signalization events mediated by arbuscules could work both ways, whether by inducing/repressing genes depending on its role in the regulation of root water relations. Another important agent of osmotic adjustment in BAT477 genotype during drought, the hydrophilic protein LEA5 (*Late embryogenesis abundant*) (Recchia et al., 2013), underwent differential regulation in cortical cell, with a -1.39-fold down-regulation in ctx+ cells in relation ctx-, and a 1.59-fold up-regulation in relation to ctrl (**Figure 9**). Since some exclusive LEA proteins related transcripts were found differentially regulated between non-inoculated conditions (Myc-/Ctrl vs. Myc-/Strss) (**Supplementary Table S4**), with a down-regulation in water deficit treated roots, the accumulation of *LEA5* transcripts in cortical tissues would offer a potential mechanism for drought tolerance in mycorrhizal roots.

Another important molecular function in drought-stress response is the regulation mediated by the binding of TFs into the *cis*-regulatory promoter region of downstream stress-responsive genes (Joshi et al., 2016). The TF *activity* was exclusively enriched by the *Myc-/Ctrl vs. Myc-/Strss* comparison, exhibiting an over-representation of TF-related transcripts (**Figure 5B**). Six TFs were chosen for our LCM/RT-qPCR analysis and distinct patterns of up-/down-regulation between cortical cells were found, probably due to the diversity of metabolic functions they are associated with. Transcripts of *bZIP* and *bhlh95* were not amplified in ctx- cells (**Supplementary Figure S2**). *bZIP* holds a basic region/leucine zipper motif and in common bean roots were enrolled in drought tolerance (Rodriguez-Uribe and O’Connell, 2006). This gene showed the highest level of differential regulation between two samples with a 79.70-fold up-regulation in ctx+ cells in relation to ctrl. *bhlh95*, a putative circadian-clock protein, a time-keeping mechanism used to coordinate the physiology of an organism to its surrounding environment (Uno et al., 2000; Xue and Loveridge, 2004), was down-regulated by -4.75-fold in ctx+ in relation to ctl. The MADS-box TF-related *SRF* transcripts were only quantified in AM-root tissues with a -2.9-fold down-regulation in ctx+ cells in relation to ctx- (**Figure 9**). This same pattern was observed for another *MADS-box TF* gene in *M. truncatula* roots colonized by the AMF *G. intraradices* (Gaude et al., 2012), indicating the relevance of this transcripts accumulation in neighboring epidermal cells during AM colonization. The two No-apical meristem-related genes, *PvNAM4* and *PvNAM28*, both belonging to the stress-responsive superfamily of TFs NAC (NAM/Ataf/CUC) (Puranik et al., 2012; Recchia et al., 2013), were detected in the three cell types (**Supplementary Figure S2**), although differential analysis was only possible for *PvNAM4* with a similar pattern of regulation of *LEA5*. The *ZFHD*, that encodes a zinc-finger homo-domain dimerization protein associated with the regulation of plant development, was the only one detected in all cell types analyzed as up-regulated in the ctx+ cells in relation to both ctx- (2.1-fold) and ctrl (1.4-fold) samples (**Figure 9**).

Genes putatively associated with nucleolar pre-rRNA processing (*Utp23*) (Rempola et al., 2006), ribosome targeting to the ER membrane (*PvNAC4*) (Wiedmann and Prehn, 1999), Ran-GTP accumulation around chromosomes and coordination of mitotic spindle formation (*RCC1*) (Makde et al., 2011), and transposase activity (*hAT*) (Rubin et al., 2001) were also tested (**Figure 9**) providing additional insights about the range of arbuscular mycorrhizal impact over cellular metabolism covering localized protein biosynthesis, mediated by ribosomal assembly and transport, and regulation of cell cycle and DNA recombination.

The *CKX* transcript, a cytokinin dehydrogenase, was not amplified in ctx- cells (**Supplementary Figure S2**), with a 2.53-fold up-regulation in ctx+ cells in relation to ctrl (**Figure 9B**). Cosme et al. (2016) indicated that reduced CK levels in roots not only caused root and shoot growth depression, but also impaired AMF colonization and suggested that root CK might regulate the carbon availability from the roots to the fungus during stressful conditions.

Terpenoid biosynthesis occurs within specific tissues, with its regulation induced in response to herbivore feeding, attack by pathogen or abiotic stresses (Singh and Sharma, 2015). The *TPS* (terpene synthase) gene was 45.4-fold up-regulated in ctx+ cells in relation to ctx- (**Figure 9A**). This gene is potentially associated with the endogenous regulation of another important plant hormone, the brassinosteroid strigolactone, implicated in the signaling communication established between both partners during the symbiosis (Martín-Rodríguez et al., 2011). Additionally, this gene was down-regulated in ctx+ relative to ctrl, indicating a reduction in terpenoids concentration in arbusculated cells during water deficit.

The colonization process in AM plants is not a synchronized event, and after an initial step of colonization of the root cortex, secondary events of infection are initiated and the invasion process is restarted (**Figure 1D**; Foo et al., 2013). Therefore, a series of genes related to cell-wall, membrane, and cytosolic organization; apoptosis initiation; cellular division; and developmental progression are differentially regulated during the symbiosis. An α/β hydrolase, *ABHD*, probably enrolled in cell-wall organization or biogenesis, was not amplified in ctx- cells (**Supplementary Figure S2**), with a -2.43-fold down-regulation in ctx+ cells in relation to the ctrl (**Figure 9B**). *Apg9*, related to addressing cytosolic compounds to autophagosomes (Noda et al., 2000), was up-regulated in ctx+ cells in relation to both other cell types, ctx- and ctrl (**Figure 9**).

The fact that the spread of mycelium occurs only in the root cortex suggests that the plant may exert a modulated control over fungal proliferation, and although typical structure defense barriers such as papillae or wall appositions containing callose, phenolic compounds, or lignin are not elicited, other elements such as phenylpropanoid biosynthesis, enzymes involved in ROS scavenging, and the regulation of PR genes have been described (Hause and Fester, 2005). From the DEG associated with defense and response to pathogenicity, *NB-ARC-LRR*, a *pathogen-associated molecular patterns* (PAMP) receptor, associated to apoptosome formation and programed cell death (DeYoung and Innes, 2007), and *GDH*, a glucose-dehydrogenase belonging to the (GMC)-oxidoreductase family, a powerful biocontrol agent in plants (Kawabe et al., 2011), were both up-regulated in ctx+ cells in relation to ctrl, with *NB-ARC-LRR* also up-regulated in relation to ctx- (**Figure 9**). The *GH3*, coding for a glucan 1,3-β-glucosidase, an abundant PR2-class protein involved in cell division, trafficking of materials through plasmodesmata, abiotic stress tolerance, flower maturation, and defense against fungal pathogens (Balasubramanian et al., 2012), was amplified only in ctx+ cell (**Supplementary Figure S2**). Additionally, the lipid-transfer protein (LTP) family *LTP*, a protease inhibitor containing a LTP2 domain associated with lipid movement and solubilization between membranes, involved in a series of processes like pathogen-defense reactions and adaptation to environmental changes (Kader, 1997), was repressed in ctx+ cells in relation to ctrl, although up-regulated when compared to ctx-. These results corroborate previous observations that the process of plant-AMF recognition involves similar mechanisms related to pathogen elicitation and infection response, probably inducing a series of systemic responses which could be sufficient for a priming effect in secondary stress events (Hause and Fester, 2005).

## Conclusion

Although AMF colonization revealed no significant phenotypical alterations in common bean plants during a water deficit event, differential gene expression analyses of NGS data offered the evidences of a profound shift in the transcriptional regulation profiles of these plants that might have a considerable impact over some key metabolic pathways associated with drought response. Complementary analyses pointed aquaporin genes as potential targets for this differential regulation and that in roots, substantial AMF influence over common bean’s transcriptome profiles might occur between 48 and 72 h. After that periods, AMF influences were more intense in leaves, with eight aquaporins, out of nine, exhibiting up-regulation in water deficit treated AMF-inoculated plants in relation to non-inoculated ones. In addition, we also provide the basis for the existence of a controlled mechanism, mediated by the presence of arbuscules at root cortical cells that might exert both local and systemic influence over plants gene expression profiles.

## Author Contributions

GR, EK, and ST conceived and designed the experiments. GR, EK, and FC performed and analyzed the greenhouse water deficit assay. GR performed the RNA-Seq experiment and bioinformatic analysis. GR and FC performed light-microscopy and LCM analysis. GR, EK, and DC were responsible for the design, execution, and analysis of all RT-qPCR experiments. All authors were involved in this article writing. ST revised the paper critically for important intellectual content. GR, EK, FC, DC, and ST read and approved the final version of the manuscript.

## Conflict of Interest Statement

The authors declare that the research was conducted in the absence of any commercial or financial relationships that could be construed as a potential conflict of interest.

## Footnotes

1 http://imagej.nih.gov/ij

2 https://cran.r-project.org/web/packages/laercio/index.html

3 https://github.com/ibest/seqyclean

4 http://www.ncbi.nlm.nih.gov/VecScreen/UniVec.html

5 http://phytozome.jgi.doe.gov/pz/portal.html

6 http://www.ncbi.nlm.nih.gov/sra/SRP070570

7 http://jgi.doe.gov/

8 http://www-huber.embl.de/users/anders/HTSeq/doc/index.html

9 http://www.ncbi.nlm.nih.gov/books/NBK52640/

10 www.ncbi.nlm.nih.gov, accessed in: 12/11/2013

11 www.geneontology.org

12 http://bioinfo.ut.ee/primer3-0.4.0/

13 www.premierbiosoft.com/netprimer

## Supplementary Material

FIGURE S1 Soil moisture content and root arbuscular mycorrhizal fungi colonization rate. **(a)** Moisture content (%) of soil samples collected from pots containing plants after at the beginning of irrigation interruption (0), and after 24, 48, 72, and 96 h of water deprivation. **(b)** Average AMF colonization rates (%) of fresh roots harvested from the four biological samples from each treatment. Myc+/Stress, AM plants under 96 h of water deficit; Myc+/Ctrl, AM plants under regular irrigation control; Myc-/Stress, no-AM plants under 96 h of water deficit; Myc-/Ctrl, no-AM plants under regular irrigation control.

FIGURE S2 Amplification products. **(a)** Amplification products obtained after transcripts amplification through RT-qPCR. **(b)** Amplification stability test for the housekeeping gene *skip16* applied as an internal normalizer in relative expression analysis. **(c)** Amplification of AMF1/AMF2 primers (endogenous control) for each cell-type considering biological triplicates. Legends: ctx+, arbusculated root cortical cells from Myc+/Strss plants; ctx-, non-arbusculated neighboring root cortical cells from Myc+/Strss plants; ctrl, root cortical cells from Myc-/Strss plants. The absence of bands indicate no-amplification of a given transcript.

TABLE S1 General RNA-Seq statistics. Number of raw reads obtained after paired-end sequencing. Total amount (%) of reads after quality-check and filtration of raw data. Number of reads mapped using the *P. vulgaris* reference genome v.11.0. Mapping Statistics. NCBI’s SRA Submission ID.

TABLE S2 Primers designed for cell-type RT-qPCR analysis. Primers name, amplicon gene annotation according to *P. vulgaris* public database, nucleotide sequence, annealing temperature (°C), GC(%) content, and amplicon size (bp).

TABLE S3 Complete set of DEGs obtained after treatments comparison. Four statistical comparisons were obtained and are shown in separated sheets: Myc+/Ctrl × Myc+/Strss (AMF-inoculated, control vs. water deficit); Myc-/Ctrl × Myc-/Strss (non-inoculated, control vs. water deficit); Myc-/Strss × Myc+/Strss (water deficit, AMF-inoculated vs. non-inoculated); Myc-/Ctrl vs. Myc+/Ctrl (control, AMF-inoculated vs. non-inoculated) (separated in different spreadsheets). DEGs were annotated following *P. vulgaris* reference genome (v.11.0). Relative gene expression values are indicated in log2FoldChange. DEGs are ordered according to adjusted *p*-value at a false-discovery rate (FRD) <0.05 (FDR *p*-value).

TABLE S4 List of DEGs that are unique for each statistical comparison: Myc+/Ctrl × Myc+/Strss (AMF-inoculated, control vs. water deficit); Myc-/Ctrl × Myc-/Strss (non-inoculated, control vs. water deficit); Myc-/Strss × Myc+/Strss (water deficit, AMF-inoculated vs. non-inoculated); Myc-/Ctrl vs. Myc+/Ctrl (control, AMF-inoculated vs. non-inoculated) (separated in different spreadsheets). Relative gene expression values are indicated in log_2_FoldChange. DEGs are ordered according to adjusted *p*-value at a FRD <0.05 (FDR *p*-value).

TABLE S5 Enrichment analysis of ontology terms. Test set: list of DEGs retrieved after the statistical comparisons: Myc+/Ctrl × Myc+/Strss (AMF-inoculated, control vs. water deficit); Myc-/Ctrl × Myc-/Strss (non-inoculated, control vs. water deficit); Myc-/Ctrl vs. Myc+/Ctrl (control, AMF-inoculated vs. non-inoculated) (separated in different spreadsheets). Reference set: total set of *Phaseolus vulgaris* transcripts. Enriched ontology terms are defined according to Gene Ontology ids. C, cellular component; F, molecular function; P, biological process. Data are organized according to *p*-value adjusted with a FDR <0.01.

TABLE S6 Relative expression levels of the 41 *P. vulgaris* aquaporin-related genes after differential analysis of RNA-Seq data. Two statistical comparisons are provided: Myc+/Ctrl × Myc+/Strss (AMF-inoculated, control vs. water deficit); Myc-/Ctrl × Myc-/Strss (non-inoculated, control vs. water deficit) (separated in different spreadsheets). Genes highlighted in red are up-regulated at control conditions and in green are down-regulated. Genes that are not-highlighted genes did not show statistical significant changes (*p*-value at a FRD <0.05). Relative gene expression values are indicated in log_2_FoldChange.
