# Supplementary figures and images for: Arbuscular Mycorrhizal Symbiosis Leads to Differential Regulation of Drought-Responsive Genes in Tissue-Specific Root Cells of Common Bean
Source: Front Microbiol. 2018 Jun 21;9:1339. doi: 10.3389/fmicb.2018.01339 (PMC6036286; doi:10.3389/fmicb.2018.01339)

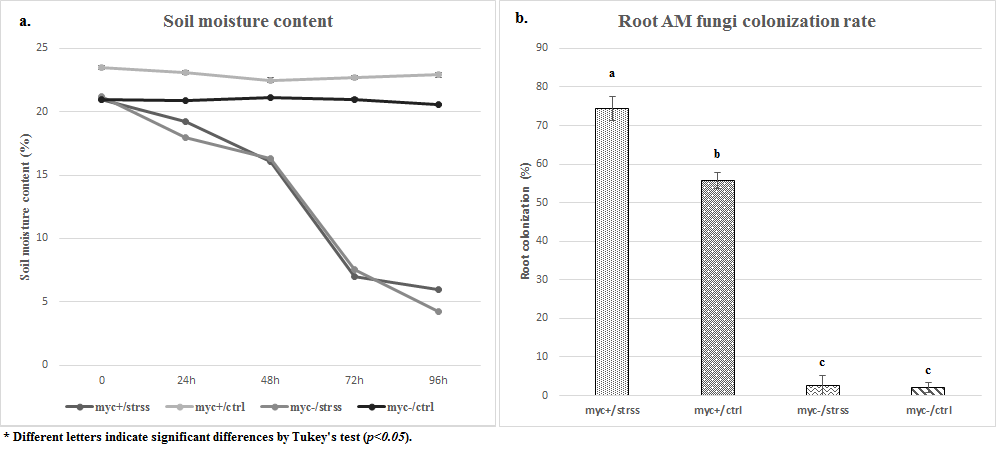

Supplement: FIGURE S1 — Soil moisture content and root arbuscular mycorrhizal fungi colonization rate. (a) Moisture content (%) of soil samples collected from pots containing plants after at the beginning of irrigation interruption (0), and after 24, 48, 72, and 96 h of water deprivation. (b) Average AMF colonization rates (%) of fresh roots harvested from the four biological samples from each treatment. Myc+/Stress, AM plants under 96 h of water deficit; Myc+/Ctrl, AM plants under regular irrigation control; Myc-/Stress, no-AM plants under 96 h of water deficit; Myc-/Ctrl, no-AM plants under regular irrigation control. [file Image_1.TIF]

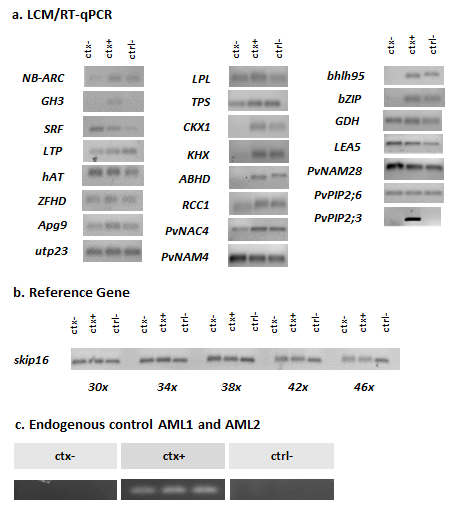

Supplement: FIGURE S2 — Amplification products. (a) Amplification products obtained after transcripts amplification through RT-qPCR. (b) Amplification stability test for the housekeeping gene skip16 applied as an internal normalizer in relative expression analysis. (c) Amplification of AMF1/AMF2 primers (endogenous control) for each cell-type considering biological triplicates. Legends: ctx+, arbusculated root cortical cells from Myc+/Strss plants; ctx-, non-arbusculated neighboring root cortical cells from Myc+/Strss plants; ctrl, root cortical cells from Myc-/Strss plants. The absence of bands indicate no-amplification of a given transcript. [file Image_2.TIF]
