# Supplementary material for: Arbuscular Mycorrhizal Symbiosis Leads to Differential Regulation of Drought-Responsive Genes in Tissue-Specific Root Cells of Common Bean
Source: Front Microbiol. 2018 Jun 21;9:1339. doi: 10.3389/fmicb.2018.01339 (PMC6036286; doi:10.3389/fmicb.2018.01339)
Supplement: TABLE S2 — Primers designed for cell-type RT-qPCR analysis. Primers name, amplicon gene annotation according to P. vulgaris public database, nucleotide sequence, annealing temperature (°C), GC(%) content, and amplicon size (bp). [file Table_2.DOCX]

**Table S2.** Primers designed for RT-qPCR analyses. Primers name, amplicon gene annotation according to P. vulgaris public database, nucleotide sequence, aneling temperature (˚C), GC(%) content and amplicon size (bp).

**Aquaporin-related *P. vulgaris* transcripts**

| **Gene name** | **Locus** | **Primer** | **Tm (°C)** | **GC%** | **(pb)** |
| --- | --- | --- | --- | --- | --- |
| PvTIP1;1 | Phvul.001G181100 | F: CAACAAGCTTACCGACAACG | 59,39 | 50 | 198 |
|  |  | R: CTGGGCGATGATGTAGACG | 60,23 | 57,89 |  |
| PvPIP1;5 | Phvul.010G004500 | F: GAGACAACCCATTGGTACGG | 60,23 | 55 | 175 |
|  |  | R: TGACGGTTAAGACGGTGATG | 59,57 | 50 |  |
| PvPIP1;2 | Phvul.008G226000 | F: ATTAGGTGGTGCATGTGTCG | 60,09 | 50 | 180 |
|  |  | R:CAGCCGTACATCACACATAGAG | 60,04 | 50 |  |
| PvPIP1;1 | Phvul.001G241200 | F: GTGGGTAAAAGAACCAGACG | 60,06 | 50 | 153 |
|  |  | R: CACATCGCTCATGGTCATC | 60,07 | 50 |  |
| PvPIP2;5 | Phvul.011G079300 | F: CTCCAACAAGGTCATGGTGT | 60,11 | 55 | 150 |
|  |  | R: CCCACAACAACTGTAGCTGA | 60,05 | 45 |  |
| PvPIP1;3 | Phvul.002G290400 | F: GGTACAGCAGCACAAGGTGA | 59,9 | 55 | 167 |
|  |  | R: CCTGTTGACACCCATGACAG | 60 | 55 |  |
| PvPIP2;3 | Phvul.007G094600 | F: CTTCCCATTGGATTTGCTGT | 60,14 | 50 | 161 |
|  |  | R: CCGGGGCAAAAGATACACTA | 59,95 | 50 |  |
| PvPIP2;1 | Phvul.004G082600 | F: TTGTGAGAGCGAGATCGTTG | 59,93 | 50 | 181 |
|  |  | R: TGAGGAACTTGCGGATCTCT | 59,95 | 50 |  |
| PvPIP2;6 | Phvul.001G177000 | F: CGCAGAGATTATTGGCACCT | 60,24 | 50 | 149 |
|  |  | R: ACAGGGATGGTGGCTAAGTG | 59,99 | 55 |  |

**LCM/RT-qPCR – root cortical cells relative gene expression analysis**

| ***Primer*** | **Putative Annotation** | **Sequence** | **Tm (°C)** | **GC%** |  | **bp** |
| --- | --- | --- | --- | --- | --- | --- |
| *NB-ARC-LRR* | Apoptotic ATPase | F:AGGATGTGCAAGGTCTGAGA | 59,91 | 45 |  | 168 |
|  |  | R:GGCTCTCAAATCCATCCAACA | 59.68 | 45 |  |  |
| *GH3* | Glucan 1,3-βGlucosidase | F:TTCAGGCTGCACTTGATTGG | 58,75 | 50 |  | 163 |
|  |  | R:AAATCACAGGTCCCAGCAGA | 58,9 | 50 |  |  |
| *SRF* | SRF-type (MADS-box) | F:GGAGGGGAAGAGTGGAGTTG | 59,38 | 60 |  | 164 |
|  |  | R:CTCATAAAGCTTGCCACGGT | 58,55 | 50 |  |  |
| *PvNAC4* | PvNAC4 | F:AGCAGTTCAGGATGCCAGAT | 59.83 | 50 |  | 198 |
|  |  | R:TGTCTCCGTCGTGAGTCTTG | 60.02 | 55 |  |  |
| *hAT* | Transposase hAT | F:TGGATGTGAGCGAAATTGGAG | 58,64 | 47,6 |  | 155 |
|  |  | R:CATCAAAGGGAAGAGCAACTGT | 58,85 | 58,85 |  |  |
| *LTP* | Protease inhibitor | F:CCAAATCCTTAGTGACGCCA | 57,89 | 50 |  | 186 |
|  |  | R:TGACCCTGCCAACATCAAAG | 58,38 | 50 |  |  |
| *ABHD* | α/β hydrolase | F:GCTGATGTATGTGAGCCCTTG | 59,05 | 52,38 |  | 165 |
|  |  | R:ACTGGTGGAAGCACTATCTGT | 58,74 | 47,62 |  |  |
| *Utp23* | rRNA pre-processing | F:ACAGTCGTTTCGGTTTTAGGG | 60.16 | 55 |  | 180 |
|  |  | R:CGCCGCCTTCGTATGTTTAG | 59.93 | 50 |  |  |
| *bhlh95* | bHLH95 | F:GAAGCGAAATCATGGGGAGC | 59,3 | 55 |  | 162 |
|  |  | R:GGAAGATGGGGAAGCAAAGC | 59,18 | 55 |  |  |
| *PvNAM28* | NAM28 | F:GGTTTAGGTTCCACCCCACT | 60.09 | 55 |  | 123 |
|  |  | R:CCCAAGGTTCGCACTTGTAT | 59.99 | 50 |  |  |
| *Apg9* | Autophagy protein 9 | F:CAATCACCGAACACCAGACA | 60.16 | 45 |  | 159 |
|  |  | R:GGGCTGCAATAAACATCACCA | 59.39 | 45 |  |  |
| *ZFHD* | Zinc fingerHD | F:AGGAGATCGACGGGGAAATC | 59.84 | 55 |  | 124 |
|  |  | R:TAAAAGGTGGTGGTGGTGGA | 60.04 | 50 |  |  |
| *bZIP* | bZIP TF | F:TTCCCCTGTTCAAAATCGCC | 58,7 | 50 |  | 175 |
|  |  | R:AGGAGGGAAGTTGTGTAGCC | 59 | 55 |  |  |
| *RCC1* | Regulation chrm condensation | F:GTTTCCGCCGTGTTGTTACT | 59,1 | 50 |  | 167 |
|  |  | R:GAGGGAAAGGCTCTGGAGTT | 59 | 55 |  |  |
| *LPL* | Lipase | F:TGTCGGGGAGCAATGGAAAT | 59,7 | 50 |  | 156 |
|  |  | R:CGATGACTCTGGGCAAGTTG | 58,9 | 55 |  |  |
| *CKX1* | Cytokinin dehydrogenase | F:GGAGGGGCATTATGGGAAGA | 58,85 | 55 |  | 193 |
|  |  | R: CGCCTTTGCCTGTTACAACT | 59,05 | 50 |  |  |
| *GDH* | Glucose dehydrogenase | F:AGGGTAGCTTCAGGTTTGGA | 58,26 | 50 |  | 177 |
|  |  | R:CACAGAGAGCAGAGAGGGATT | 58,89 | 52,38 |  |  |
| *TPS* | Terpene synthase | F:ACCACCGGATCACAGAAGAG | 59,1 | 55 |  | 182 |
|  |  | R:ACCCTCTTCACTAGCACCAC | 59,02 | 55 |  |  |
| *KHX* | K+/H+ antiporter | F:AAAAGCAACAGCACGGACTC | 59,34 | 50 |  | 172 |
|  |  | R:CAATGGGACCAAGTTCAGGA | 57,42 | 50 |  |  |
| *PvNAM4* | NAM4 | F:AATGAAGGGTCATTCGCAAC | 59.94 | 45 |  | 132 |
|  |  | R:GAGCTGAGAGGCAACCAAAC | 60 | 55 |  |  |
| *LEA5* | LEA5 | F:CTGTTCATCGGCGAGGTTAT | 60,1 | 50 |  | 183 |
|  |  | R:TCATGGGCCTGTAGTCACCT | 60,53 | 55 |  |  |
| *PvPIP2,3* | PIP2,3 | F:CTTCCCATTGGATTTGCTGT | 60,14 | 50 |  | 161 |
|  |  | R:CCGGGGCAAAAGATACACTA | 59,95 | 50 |  |  |
| *PvPIP2,5* | PIP2,5 | F:CGCAGAGATTATTGGCACCT | 60,24 | 50 |  | 149 |
|  |  | R:ACAGGGATGGTGGCTAAGTG | 60 | 55 |  |  |
